# A quantitative analysis on the effects of critical factors limiting the effectiveness of species conservation in future time

**DOI:** 10.1002/ece3.3788

**Published:** 2018-02-24

**Authors:** Diogo Alagador, Jorge Orestes Cerdeira

**Affiliations:** ^1^ CIBIO/InBio‐UE: Centro de Investigação em Biodiversidade e Recursos Genéticos Universidade de Évora Évora Portugal; ^2^ Departamento de Matemática Faculdade de Ciências e Tecnologia Universidade NOVA de Lisboa Caparica Portugal; ^3^ Centro de Matemática e Aplicações Faculdade de Ciências e Tecnologia Universidade NOVA de Lisboa Caparica Portugal

**Keywords:** climate change, conservation planning, cost effectiveness, optimization, resource allocation, species distribution

## Abstract

The effectiveness of conservation plans depends on environmental, ecological, and socioeconomic factors. Global change makes conservation decisions even more challenging. Among others, the components of most concern in modern‐day conservation assessments are as follows: the magnitude of climate and land‐use changes; species dispersal abilities; competition with harmful socioeconomic activities for land use; the number of threatened species to consider; and, relatedly, the available budget to act. Here, we provide a unified framework that quantifies the relative effects of those factors on conservation. We conducted an area‐scheduling work plan in order to identify sets of areas along time in which the persistence expectancies of species are optimized. The approach was illustrated using data of potential distribution of ten nonvolant mammal species in Iberia Peninsula from current time up to 2080. Analyses were conducted considering possible setups among the factors that are likely to critically impact conservation success: three climate/land‐use scenarios; four species’ dispersal kernel curves; six land‐use layer types; and two planning designs, in which assessments were made independently for each species, or joining all species in a single plan. We identified areas for an array of investments levels capable to circumvent the spatial conflicts with socioeconomic activities. The effect of each factor on the estimated species persistence scores was assessed using linear mixed models. Our results evidence that conservation success is highly reliant on the resources available to abate land‐use conflicts. Nonetheless, under the same investment levels, planning design and climate change were the factors that most shaped species persistence scores. The persistence of five species was especially affected by the sole effect of planning design and consequently, larger conservation investments may retard climatic debts. For three species, the negative effects of a changing climate and of multiple‐species planning designs added up, making these species especially at risk. Integrated assessments of the factors most likely to limit species persistence are pivotal to achieve effectiveness.

## 1 INTRODUCTION

Climate change is accelerating species loss, and by the end of this century, as many as one in six species will be at risk of extinction (Urban, 2015). To avert this outcome, spatial conservation planning must be dramatically revised (Araújo, Alagador, Cabeza, Nogués‐Bravo, & Thuiller, 2011; Lawler, 2009; Moritz & Agudo, 2013; Oliver, Smithers, Beale, & Watts, 2016). Climate change will make biodiversity conservation more challenging and expensive (Busch et al., 2012; Shaw, Klausmeyer, Cameron, Mackenzie, & Roehrdanz, 2012; Wise, Reyers, Guo, Midgley, & De Lange, 2012), and as such, given the scarcity of the financial resources that are typically available conservation must act cost effectively. The integration of the requirements of multiple species (MS) within planning designs is the first step toward efficient decision making (Nicholson & Possingham, 2006; Poiani, Richter, Anderson, & Richter, 2000). In MS plans, the efforts needed to fulfill the requirements of one species are shared with the requirements of other species. These requirements are based on estimates of adequate levels of species representation in conservation areas in order to encourage species persistence (Carwardine, Klein, Wilson, Pressey, & Possingham, 2009; Pressey, Cowling, & Rouget, 2003). However, implementing MS conservation plans with tight budgets implies trade‐offs among species such that the targets initially established for some species might not be fully satisfied (Van Teeffelen, Cabeza, Pöyry, Raatikainen, & Kuussaari, 2008). Budgetary restrictions are also inflated by the need to act in lands degraded by socioeconomic uses and by the increasing number of species requiring conservation interventions in a highly dynamic world.

The effectiveness of conservation plans depends heavily on reaching species‐specific targets. Typically, targets define the range size, abundance, or genetic variability of a species that must be represented in conservation areas for it to persist (Pressey et al., 2003). However, setting representation targets in MS conservation plans is not a trivial task (Alagador & Cerdeira, 2017). Setting too ambitious targets for some species results in less resources being available to conserve the remaining species, and these trade‐offs should be assessed (Di Minin & Moilanen, 2012; Moilanen & Arponen, 2011).

The current “Anthropocene Era” is characterized by the pervasive use of land by humans and a concurrent, significant increase in the pace of climate change (Jantz et al., 2015; Pouzols et al., 2014). The adaptive movements of many species toward new suitable habitats are hindered by the negative effects of socioeconomic activities on natural environments, which subjects species (especially those with very limited dispersal abilities) to unavoidable climate stress and, ultimately, to extinction debt (Engler & Guisan, 2009; Higgins & Richardson, 1999; Hof et al., 2012). Furthermore, the expansion of traditional socioeconomic activities, which increases land prices through market forces, and the increased efforts to restore degraded habitats make conservation plans more expensive to implement (Balmford, Gaston, Rodrigues, & James, 2000).

In this melting pot of threats to species persistence, a unified quantitative assessment to identify the factors that are most likely to limit species conservation efforts in future time is required. Here, we make such assessment. Specifically, we confront the sensitivity of species persistence in spatial conservation plans to environmental (i.e., climate and land‐use scenarios), ecological (i.e., species dispersal abilities), and socioeconomic (i.e., land‐use occupancy) factors. Additionally, because the availability of financial resources may conduct to decisions on the number of species to be protected in a single conservation plan, we tested the effect of choosing between single‐species (SS) versus MS plans. We illustrate the approach using comprehensive data on the distributions of ten nonvolant mammal species of conservation concern on the Iberian Peninsula up to 2080, using a target‐based spatial conservation model guided by species persistence scores (Alagador, Cerdeira, & Araújo, 2016). For each species, we selected a reference scenario consisting of a dedicated (ideal) SS conservation plan, and we ran it for distinct climate/land‐use projections, different dispersal assumptions, and distinct land uses (i.e., socioeconomic activities). Analyses were performed for a range of scenarios with conservation areas of increasing size and consequently increasing levels of conflict with socioeconomic activities (thus, with increase dependency on larger budgets for area acquisition). The persistence of each species under each of these SS plans was used as targets in the respective MS plans (in which the gaps between the optimized and the targeted species persistence scores are minimized). We assessed the sensitivity of the persistence scores obtained for each species to variations in each of the tested factors to estimate the additive effect of each factor on species conservation effectiveness.

## 2 METHODS

### 2.1 Species data

We considered a sample of ten terrestrial, nonvolant mammal species occurring on the Iberian Peninsula (hereafter Iberia) that are listed as threatened (i.e., vulnerable, endangered, or critically endangered) in the IUCN Red Lists of Portugal (Cabral et al., 2005) and Spain (Dirección general para la biodiversidad 2007) (Table 1).

**Table 1:** The species analyzed including scientific name, status under the Portuguese (RL Portugal) and Spanish (RL Spain) Red Lists (IUCN categories: CR: critically endangered; EN: endangered; VU: vulnerable; NT: near threatened; LC: least concern; DD: data deficient); maximum dispersal distance in a 30‐year time‐period (*D*_max_), and the expected number of occupied cells in the baseline period (Baseline) as obtained by an ensemble of bioclimatic niche models (only considering cells with confirmed presences)

Species	Abrev.	RL Portugal	RL Spain	*D*_max_ (km)	Baseline
*Arvicola sapidus*	*Asa*	LC	VU	20	138.2
*Galemys pyrenaicus*	*Gpy*	VU	EN	10	40.7
*Microtus cabrerae*	*Mca*	VU	VU	10	7.5
*Oryctolagus cuniculus*	*Ocu*	NT	VU	65	1081.7
*Mustela erminea*	*Mer*	DD	VU	31	410.0
*Mustela lutreola*	*Mlu*	–	EN	115	56.1
*Capra pyrenaica*	*Cpy*	CR	VU	160	228.0
*Felix sylvestris*	*Fsy*	VU	VU	200	11.3
*Canis lupus*	*Clu*	EN	NT	200	492.6
*Ursus arctos*	*Uar*	–	CR	200	72.5

Species distribution data for a baseline time‐period (1990) and for three future time‐periods (2020, 2050, and 2080) were obtained from an ensemble of bioclimatic niche models (see Appendix S1) projected in a 10’ squares gridded map of Iberia (2,310 grid cells). We assumed three future climate scenarios: one simulating the maintenance of current climatic conditions and land‐use patterns (nonchanging, NC) and two following the A1FI and B1 IPCC AR4 scenarios (Nakicenovic et al., 2000). Although more recent climate projections exist after IPCC AR5, we opted to use the AR4 as it links with the most resolute land‐use projections for Europe (see spatial conflicts with socioeconomic activities). Moreover, there exists considerable analytical evidence that there are no major distinctions in making species distribution modeling from both sources (Murray et al., 2017; Ring et al., online first; Wright, Schwartz, Hijmans, & Shaffer, 2016). Each mapped grid cell, *i* (henceforth, cell), records the local climatic suitability for each species, *s*, which ranges from zero‐to‐one, for each time‐period, *t* (${\text{po}}_{s}^{\mathrm{it}}$). For the baseline period, *t *=* *1, the species suitability scores for cells with no species records were settled to zero (i.e., ${\text{po}}_{s}^{i1}\;=\;0$).

Dispersal ability is an essential factor that affects the capacity of species to adapt to changing climates. Given that long‐distance dispersal is an important factor in the success of species range expansions, we defined a conservative maximum dispersal distance for a 30‐year time‐period, *D*_max_, for computational efficiency. Due to the scarcity of empirical data on species dispersal capabilities in spatiotemporal contexts, we used allometric relationships (based on adult body weight and generation time) to define *D*_max_ (Schloss, Nuñez, & Lawler, 2012) (see Appendix S1). For each species, we tested four dispersal curves to estimate the probability of dispersal success based on the geographic distance between a source, *i,* and establishment cells, *j* (${\text{pd}}_{s}^{\mathrm{ij}}$). We chose scenarios simulating a nondispersal case and three dispersal curves for which the probability of successful dispersal to *D*_max_ was 5, 10, and 15%.

Given the large area covered by each cell, we considered every cell to be potentially usable by all dispersing species; therefore, we considered a linear geographic distance acceptable to emulate dispersal success rates. We excluded the cells showing high anthropogenic disturbance at the baseline time from the analysis. Disturbance was measured by averaging the 1‐km × 1‐km 1995–2004 Human Footprint Index (Sanderson et al., 2002), and cells with average values >60 (a value characterizing urban landscapes) were assumed unsuitable for conservation along all the analyzed time horizons.

### 2.2 Climate change corridors (CCCs)

The scientific literature on conservation planning is prolific on spatial conservation models. However, few have been dedicatedly developed to integrate both the environmental and socioeconomic dynamics (but see, Alagador et al., 2016) and to provide researchers with an area scheduling plan depicting spatiotemporal conservation units in which species’ persistence is evaluated. In this regard, we used the concept of climate change corridors, CCCs (sensu Alagador, Cerdeira, & Araújo, 2014; originally introduced by Williams et al., 2005 and improved by Phillips, Williams, Midgley, & Aaron, 2008), to optimize future conservation decisions. A CCC defines a sequence of time‐ordered cells that are occupied by a species through time. The probability of a species, *s*, to persist in a given corridor, *c*, formed by cells *u1, u2,…, uT* that are selected for the time‐periods *t *=* *1,2,…,*T* is as follows:

(1) $$P_{s}^{c}\;=\;po_{s}^{u1}\;\times \;pd_{s}^{u1,u2}\;\times \;po_{s}^{u2}\;\times \;\ldots \;\times \;po_{s}^{u(T-1)}\;\times \;pd_{s}^{u(T-1),uT}\;\times \;po_{s}^{\mathrm{uT}}$$

The persistence of a species, *s*, in a set of CCCs, *C*, is the maximum sum of the persistence scores for the set of independent corridors in *C*; a set of corridors is independent if no two corridors intersect (i.e., if the same cell is not to be used by both corridors in respect to the same time‐period). The concept of independent corridors was introduced as a convenient property to mitigate the spread of unforeseen negative contagious events.

The identification of effective CCCs satisfies distinct conservation requirements (Alagador et al., 2016) and provides both spatial (where to prioritize) and temporal (when to prioritize) information for planners. We implemented two distinct CCC selection models. One model (*maxPers*) simulates an ideal scenario in which conservation planning is undertaken independently for each species with the objective of maximizing the persistence of the species within the CCCs selected under a given investment constraint, *B* (i.e., amount of area covered or, equivalently, a given level of socioeconomic conflict; see below). The other model (*minShortfall*) was developed for the implementation of MS planning, assuming explicit conservation targets for each species (in this case persistence scores). The model searches for the CCCs in which the summed shortfalls (among species) of the persistence scores in the CCCs to the targeted scores are minimized under the investment constraint, *B*. To circumvent biased selections of CCCs when species persistence scores vary by several orders of magnitude, shortfalls were formulated as proportional deviances to the persistence targets (see Appendix S1 for the mathematical details).

Species persistence scores (Equation (1)) do not deliver directly interpretable information (i.e., probability of persistence), but their scalability might be assessed when confronted with extreme reference scenarios. A zero‐persistence corridor for a species, *s*, is made by at least a “nonfunctional cell,” *i*, where the probability of the species occurring at some time‐period, *t*, is zero (${\text{po}}_{s}^{\mathrm{it}}$ = 0) or by a “nonfunctional dispersal” in which the colonization of cell *j* (from cell *i*) is improbable (${\text{pd}}_{s}^{\mathrm{ij}}$). Likewise, a corridor with a high probability of persistence for a species is made of cells with the highest suitability in all time‐periods and across which that species disperses with great success. In a solution, the final persistence associated with each species results from summing the persistence scores among the selected independent CCCs. Thus, optimal solutions correspond to sets of corridors where the suitability for and dispersal of species are as large as possible. Given the discrepancies in species prevalence (i.e., number of records) in the bioclimatic niche modeling, the range of possible occurrence probabilities might differ between species (see Appendix S1, Table S1), thus resulting in persistence scores that vary by different orders of magnitude.

Given the mathematical complexity of the problems herein formulated, we carried out a preprocessing to restrict the analysis to a subset of corridors that were likely to deliver an optimal result (Alagador et al., 2016). After several testing runs, we identified a set of 2,000 corridors for each species for selection (see Appendix S1, Figures S5 and S6).

### 2.3 Spatial conflicts with socioeconomic activities

We used projections of land‐use/land‐cover change through time from the ALARM project dataset (Settele, Carter, KüHn, Spangenberg, & Sykes, 2012) to quantify potential conflicts that may arise between conservation planning and the main socioeconomic activities in Iberia. We aligned the GRAS and the SEDG land‐use/land‐cover ALARM storylines with the A1FI and B1 climate scenarios, respectively. These data present the land class occupancy in each cell (i.e., proportional area), and we reclassified these data to represent the main socioeconomic uses of land in Iberia (agriculture, pastures, forestry, and urban area) (see Appendix S1 for details). We then assigned each socioeconomic activity in a cell, *i*, an opportunity cost for current and future time‐periods that inform about the foregone market revenues resulting from local conservation requirements (cost_*i*_) (see Appendix S1 for details). We additionally analyzed two representations of land use in Iberia, resulting in: a uniform opportunity cost among all Iberia and an opportunity cost resulting from all the socioeconomic activities in analysis (total conflict).

We carried out analyses assuming a sequence of 400 budgets, *B*, for the identification of CCCs under increasing levels of conflict. We tested *B* values starting at four (i.e., the minimum cost needed to define a single four time‐period CCC using the uniform cost layer) with four‐unit step increments (*B *=* *4, 8, 12, …, 1,600).

### 2.4 The analytical framework

**Figure 1:**
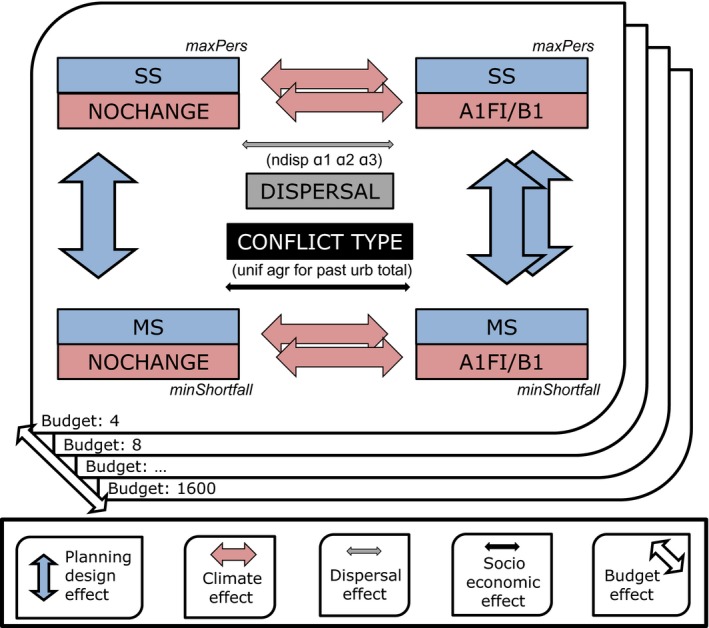
Methodological framework for quantification of the effects of climate, planning design, species dispersal, socio economic conflicts, and budget available on the persistence of species exposed to dynamic environments. SS and MS refer to single‐ and multiple‐species planning designs, respectively, in which species persistence scores are optimized using the *maxPers* and *minShortfall* model variants. Analyses were performed comparing the no‐changing (NOCHANGE) climate scenario with the A1FI and B1 climate scenarios, using distinct species dispersal abilities (ndisp: non‐dispersal and α1, α2, α3, see Methods section), distinct types of socioeconomic activities (unif: uniform, agr: agriculture, for: forest, past: pastures, urb: urban and total conflict, see Methods section) and a range of budget values (4, 8, …, 1,600)

We ran the *maxPers* model for each of the ten species for all combinations of factors (10 species x three climate scenarios x four dispersal scenarios x six socioeconomic activities × 400 budget sizes) for a total of 288,000 runs. In each run, we recorded the optimized persistence score for each species ($P_{s}^{\mathrm{SS}}$) (see Appendix S1, Equations S9‐S14). We then used these values as the persistence targets in the *minShortfall* model with the corresponding combinations of factors (28,800 runs) (see Appendix S1, Equation S2). For each species, we recorded the persistence achieved in the CCCs selected in the MS plans (Figure 1). For each species, the effect of using a MS‐plan instead of a dedicated SS plan is then quantified as the fraction of the species persistence score obtained from a *maxPers* solution (i.e., SS planning) that remains in the solutions obtained under the *minShotfall* model (i.e., MS planning), using the same budget size.

We conducted linear mixed model analyses to estimate the effect sizes of planning design, climate, dispersal, and conflict type on the persistence of each species in the CCCs, using the 400 assessed conflict levels as a random factor and assuming only additive terms (i.e., no interactions) (see Appendix S2 for details). For each factor, *F*, we obtained its marginal persistence estimate, ${\text{Peff}}_{F}^{m}$ (i.e., where the persistence estimates of the remaining covariates are set to their mean values) and the range of persistence estimates, ${\text{Peff}}_{F}^{\Delta }$, among the varying levels of *F*. The effect size of factor *F* was estimated as Peff_F_ = ${\text{Peff}}_{F}^{\Delta }/{\text{Peff}}_{F}^{m}$.

## 3 RESULTS

**Figure 2:**
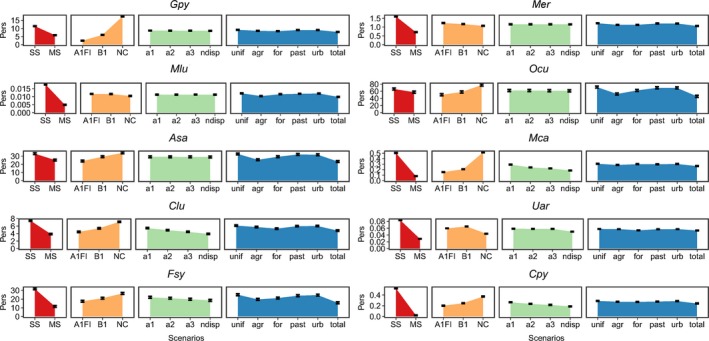
Average variations in expected species persistence scores relative to each level of the studied factors. Red polygons indicate the effect of planning design (SS: single species; MS: multiple species). Yellow polygons indicate the effect of the climate scenario (A1FI, B1, and NC: no‐change). Green polygons indicate the effect of species dispersal ability (ndisp: nondispersal; a1, a2, a3: α1, α2, and α3 kernel functions). Blue polygons indicate the effect of distinct types of socioeconomic conflicts (unif: uniform; agr: agriculture; for: forestry; past; pasture lands; urb: urban areas and total conflict). Analyses were performed for all the tested budget sizes. *Gpy: Galemys pyrenaicus; Mer: Mustela erminea; Mlu: Mustela lutreola; Ocu: Oryctolagus cuniculus; Asa: Arvicola sapidus; Mca: Microtus cabrerae; Clu: Canis lupus; Uar: Ursus arctos; Fsy: Felis sylvestris; Cpy: Capra pyrenaica*

We found that the persistence scores of species in the selected CCCs were especially sensitive to variations in the available budget, especially at their lowest values (first quartile: *R* ^2^ *m* ≪ *R* ^2^ *c,* i.e., the goodness‐of‐fit related to the fixed factors*, R* ^2^ *m*, is lower than the one related to fixed and random factors *R* ^2^ *c*, where fixed factors are the planning design, species dispersal abilities, type of socioeconomic activities, and climate change scenarios, and the random factor is the budget in use to define CCCs) (see Appendix S2, Figures S8 and S9; Appendix S3, Table S3). Among the remaining factors analyzed, planning design incited the largest variations in species persistence in the CCCs (Figure 2). For three species, the nature of conflicting activities (at the 1st budget quartile) and climate (at the 3rd and 4th budget quartiles) also influenced significantly conservation effectiveness (*Gpy, Asa,* and *Ocu*) (see Appendix S2, Figure S10; Appendix S3, Table S4).

**Figure 3:**
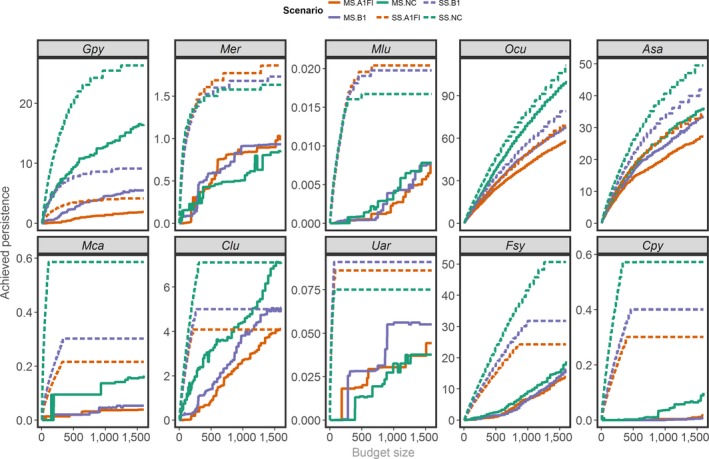
Persistence of each species within the CCCs for distinct budget sizes (or socioeconomic conflicts). Values refer to the scenario using the total conflict layer type and the α_3_ kernel function (as an example). The lines correspond to different testing cases with different climate scenarios (A1FI, B1, NC: nonchange) and planning designs (SS: single species; MS: multiple species). *Gpy: Galemys pyrenaicus; Mer: Mustela erminea; Mlu: Mustela lutreola; Ocu: Oryctolagus cuniculus; Asa: Arvicola sapidus; Mca: Microtus cabrerae; Clu: Canis lupus; Uar: Ursus arctos; Fsy: Felis sylvestris; Cpy: Capra pyrenaica*

Unsurprisingly, different species showed distinct responses to the climate scenarios. Most of the species presented the largest persistence scores under the NC scenarios, but for a few, persistence was favored under the A1FI (*Mer* and *Mlu*) or B1 (*Uar*) scenarios (Figures 2 and 3) (for the complete results, see Appendix S2, Figures S10 and S11). For three species (*Ocu, Asa,* and *Fsy*), persistence was also differentially affected depending on the socioeconomic activities assessed with the lowest persistence scores associated with a landscape represented by all the analyzed socioeconomic activities (total conflict) and the largest scores related to the uniform layer. Among all species, the additive effect of dispersal over persistence was almost negligible.

The effectiveness of the MS planning designs (compared with the corresponding SS plans) was largely related to budget size (i.e., covered area). Species persistence in the least conflicting (i.e., cheapest) CCCs obtained from the MS plans deviated significantly from the persistence scores in the corresponding SS plans (Figure 3). With larger budgets, the relative shortfalls to targets (summed among species) decayed linearly, while their respective absolute shortfall values showed a convex response (see Appendix S2, Figure S12). MS plans were especially ineffective for *Cpy*, which achieved persistence in CCCs representing 10% of the persistence targets (averaged across the tested conflict levels) (see Appendix S2, Figures S11 and S13). In contrast, the persistence of *Asa* and *Ocu* in the CCCs identified in the MS plans was highly satisfactory with very low shortfalls across all conflict levels.

**Figure 4:**
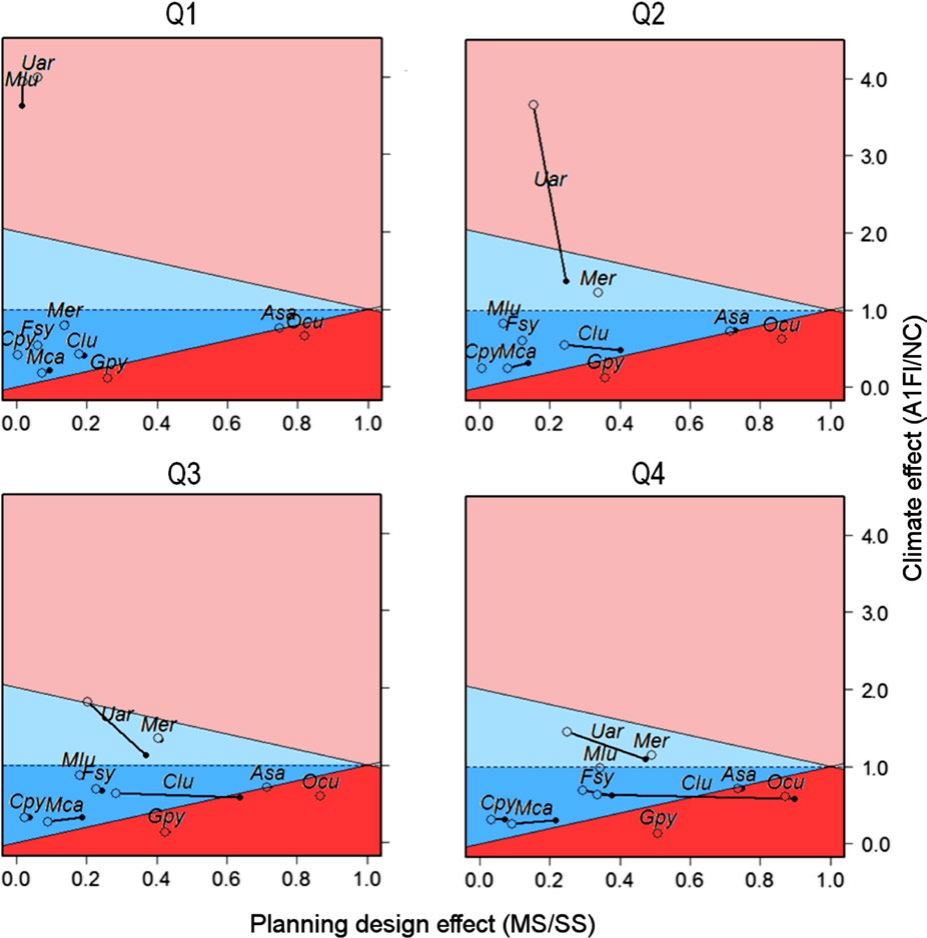
The average effect of climate (the proportional persistence expectancies for the A1FI change scenario relative to the nonchanging scenario) against the effect of planning design (proportional persistence expectancies from the SS planning design relative to those obtained from the MS designs) and the effect of dispersal rate on the persistence expectancies of the analyzed species. Average scores were taken among the 100 budget values in the 1st, 2nd, 3th, and 4th budget quartile tests. Open circles indicate the effects of climate and planning design using the α_3_ kernel function and are linked to their respective scores using the nondispersal kernel function (filled circles). Values are from testing cases using the total conflict layer (as an example). The reddish areas represent regions where climate has the largest effects on species persistence scores (light red defines the region where the positive effect of climate overpasses the negative effect of planning design; dark red defines the region where climate presents a more negative effect than planning design). The bluish areas represent regions where planning design has the largest effects on species persistence scores (light blue defines the region where the positive effect of climate is shorter than the negative effect of planning design; dark blue defines the region where climate presents a less negative effect than planning design). *Gpy: Galemys pyrenaicus; Mer: Mustela erminea; Mlu: Mustela lutreola; Ocu: Oryctolagus cuniculus; Asa: Arvicola sapidus; Mca: Microtus cabrerae; Clu: Canis lupus; Uar: Ursus arctos; Fsy: Felis sylvestris; Cpy: Capra pyrenaica*

Plotting dispersal effects against both planning design and climate effects allowed us to visualize the interdependency between these factors (Figure 4). For the smallest budgets (1st budget quartile), the effect of dispersal was negligible. With increasing budgets, the interdependency of dispersal with climate and planning design increased. Nondispersal scenarios led to more effective MS plans (values closer to one for *Clu, Mca,* and *Uar*) and an abated positive climate effect (for *Uar*) compared to any other tested dispersal scenario. For the remaining species, dispersal did not impact either planning design or climate effects.

**Figure 5:**
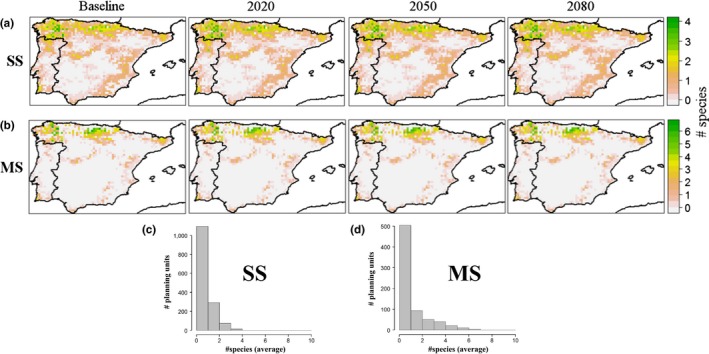
Average number of species occurring in the areas identified for CCCs (baseline period, 2020, 2050, and 2080) among the 400 tested budget values, three climate change scenarios, four dispersal success models, and six socioeconomic activities. Maps refer to (a) single‐species planning designs (SS) and (b) multiple‐species planning designs (MS). Histograms represent (c) the distributions of non‐null average species numbers among the SS and (d) MS maps

Geographically, the area accumulated by the SS solutions for all the tested budgets was approximately triple the area identified among all the MS solutions (Figure 5). For both planning designs, the prioritized areas did not change significantly with time regardless of the assessed dispersal rate. Again, planning design and climate were the factors that best explained the spatiotemporal patterns defining the CCC solutions (see Appendix S2, Figure S14; Appendix S3, Table S5).

## 4 DISCUSSION

Decision makers are rarely provided with transparent and well‐supported arguments to justify conservation investments. Our approach provides information that can be used to generate a plan to assist governments and organizations to direct funds toward the most effective actions for species conservation under changing climates (and land uses). Quantitative tools, such as the ones implemented here, are essential for integrating the (diverse) factors that are likely to drive biodiversity persistence. Thus, the use of these tools is the first step to ensure that scarce funds go as far as possible toward achieving conservation outcomes.

In our case study, the option to develop MS plans (as opposed to SS plans) was the factor on which species persistence most depended on. Accounting for a whole set of species in MS plans is counter to triage plans, in which some species are intentionally left unprotected and assumed “lost” (Bottrill et al., 2008). Planners developing MS plans recognize that species might be underprotected but also consider that the “small levels” of protection can make substantial difference as long as funding is available quickly enough to ameliorate the conservation status of species. Assuming that some species may “wait” for possible extra budgets, planners might weight species accordingly when undertaking MS approaches (e.g., by giving the highest priority to the species that need more urgent intervention).The pernicious outcomes of developing MS plans with tight spatial/budgetary constraints (when compared with SS plans) are consistent with evidence obtained elsewhere (Di Minin & Moilanen, 2012; Moilanen & Arponen, 2011) that shows the spatial relationships among species ranges, and their interactions with conservation cost largely determine the trade‐offs among species in MS plans. However, our implementation goes a step further as it extends these relationships into dynamic environmental scenarios, such as the ones framed by climate and land‐use changes. In such cases, the species with the most unique distributional patterns (and that maintain such uniqueness through time) are likely to be the most negatively impacted when grouped into a MS conservation plan (e.g., blue areas in Figure 4). When the ranges of these “unique species” overlap with areas where conflicts with socioeconomic activities are (and/or predicted to be) large, these effects may still be exacerbated.

We also found climate to be an important factor limiting conservation effectiveness. For one species (*Uar,* in Figure 4), the single effect of climate enabled it to have better persistence prospects when compared with a NC environmental scenario, regardless of the financial resources available to undertake conservation (either in SS or MS plans). For species presenting a similar response, climate is not an immediate and protection of these species may focus on averting the effects of other detrimental processes that are likely to occur with climate change (e.g., invasive species, infectious outbreaks, or altered fire regimes). For other species, the choice of planning design might constrain the potential gains from climate change (e.g., *Mer* and *Mlu* in Figure 4). The increase in the financial resources available to undertake MS plans, to design an SS plan, or, equivalently, to promote a less conflictual landscape (through regulations or agreements) might provide these species with a prospect for persistence.

Some species are likely to suffer a double negative impact from both climate change and a constrained MS planning design, but if the impacts of adopting MS plans are greatest (the dark blue area in Figure 4), the most extreme declines are potentially delayed if species are treated individually (SS plans) or if the conflicts for land with dominant socioeconomic activities are mostly addressed with conservation budgets of sufficient size (by promoting larger areas for conservation and/or by decreasing the levels of habitat disturbance derived from those socioeconomic activities). In these cases, the prospects for species persistence in MS plans approximate those obtained in SS plans with identical investment constraints. In either case, these species are likely to lose suitable climate zones and will therefore be inevitably exposed to climatic debts (Devictor et al., 2012; Urban, 2015). Increasing conservation investments might still delay the process, and in the most critical cases, huge investments in conservation management through habitat restoration assisted colonization, and green engineering (i.e., building of climatic microrefugia) have the potential to artificially make the effects of climate change positive and consequently allow the species to recover (Shoo et al., 2013). In our case study, climate change presented the largest detrimental impacts on persistence in the CCCs for *Gpy* and *Ocu* (dark red area in Figure 4), even supplanting the negative effects of MS designs. For these species, only local‐scale actions that artificially make the climate suitable for the species (i.e., microrefugia) or range‐wide actions that facilitate the relocation of species to climatically suitable areas may permit species to persist over the long‐term. Other types of interventions that do not displace species from their areas of natural occurrence will be ineffective in averting climatic debts, but if adopted, they might delay them. These interventions tend to be highly expensive and are therefore likely to deviate investment from other species with better persistence prospects (Shoo et al., 2013).

Compared with the other factors, we found that the sole effect of dispersal ability was not significant in explaining the variations in conservation effectiveness for the ten species in Iberia, but this does not mean that dispersal processes should not be considered when developing conservation plans. Increasing habitat permeability through the provision of stepping stones or biodiversity‐sensitive land‐use management, restoration of critical habitats to facilitate range shifts, and/or the artificial relocation of species to favorable areas outside their natural adaptive ranges are actions to be envisaged (Carroll et al., 2015).

The costs of conservation interventions are deeply dependent on land use and market forces (Naidoo et al., 2006). With climate change, greater financial investments will be required to either maintain or enhance the conservation statuses of species (Shaw et al., 2012; Wise et al., 2012). One mean to maximize cost effectiveness is to implement dynamic land‐use schemes (like the one we modeled herein), largely guided by the short‐ to medium‐term needs of species. Investments in areas that are no longer predicted to be of value for conservation may be directed toward other areas predicted to be of value in future time (Alagador et al., 2016), thus avoiding the accumulation of conservation areas and, consequently, reducing land‐use competition. If functional land purchase schemes exist, the release of areas may provide financial credits for conservation agencies through land banking (Armsworth, Daily, Kareiva, & Sanchirico, 2006). These decisions should be judged after considering other intangible factors that, by nature, are difficult to be modeled and that might make areas important for other purposes making social engagement with conservation easier (e.g., cultural, esthetic, historical values).

We made some simplistic assumptions in our case study. First, species persistence was evaluated based on the predicted occurrences and dispersal success of species at a broad resolution (see Equation (1)). However, planners with detailed knowledge of the (meta) population dynamics of their focal species (e.g., colonization, establishment, extinction, and expansion rates) and/or data on threats that are likely to cooccur and interact with climate change (e.g., invasive species, infectious outbreaks, and altered fire regimes) enable more precise persistence assessments for the species in CCCs to be made. Second, we assumed dispersal success dependent on geographic distance rather than functional distance. Again, planners may decide to estimate this process using a mechanistic approach that links dispersal success with habitat type or land use (Ramiadantsoa, Ovaskainen, Rybicki, & Hanski, 2015). A detailed, spatially explicit evaluation of dispersal is challenging in terms of both empirical data collection and modeling (Aben et al., 2016) and was outside the scope of our approach. Lastly, several studies have addressed the importance of developing conservation plans with cost data that best fits conservation purposes (Balmford et al., 2000; Evans et al., 2015). Here, we used land‐use occupancy to reflect potential conflicts with distinct socioeconomic activities in each cell. In general, land use is a good predictor of opportunity costs, but if available, other types of intervening conservation costs may also be integrated (e.g., acquisition and management costs) (Naidoo et al., 2006) and the results re‐evaluated.

The framework here proposed is based on mathematical programming models that may be formulated and solved using mainstream programming software (e.g., CRAN‐R, MATLAB, etc.) and free‐open‐source or commercial solvers (for a listing see, Gearhart et al., 2013). Currently, we are working on the development of a free‐accessible, stand‐alone software capable to solve the here introduced models using sizable datasets (see Appendix S4 for a very preliminary version) and therefore to provide support for planning proactive real‐world conservation under dynamic environments.

## 5 CONCLUSION

Our study introduces a framework to evaluate the addictive effects of distinct factors on the persistence of species within optimized conservation plans. We demonstrate that the impacts of climate change in conjunction with conservation underfunding, either through low‐budget SS plans, constrained MS plans, or costly socioeconomic conflicts, severely limit conservation effectiveness with varying results among species. The detailed scrutiny of these impacts may assist policy makers and planners in judging the prospects for species persistence and thus to guide their resources in order to increase conservation effectiveness under dynamic environments.

## CONFLICT OF INTERESTS

None declared.

## AUTHORS’ CONTRIBUTIONS

Both authors contributed equally in developing the conceptual and methodological designs, data evaluation, and manuscript writing. Both authors approved the final form of the manuscript.

## DATA ACCESSIBILITY

Climatic suitability data, dispersal parameterization, the socioeconomic conflict layers used in this study and a very basic and preliminary version of a software that formulates the used area scheduling models as mixed integer programming problem files (i.e., mps files) are publicly available on Figshare at https://doi.org/10.6084/m9.figshare.5700715.v1.
