# Supplementary material for: A quantitative analysis on the effects of critical factors limiting the effectiveness of species conservation in future time
Source: Ecol Evol. 2018 Feb 24;8(6):3457–67. doi: 10.1002/ece3.3788 (PMC5869367; doi:10.1002/ece3.3788)
Supplement: Supplementary file 1 [file ECE3-8-3457-s001.pdf]

# SUPPLEMENTARY MATERIAL

## APPENDIX 1

## CONTENTS


1. Bioclimatic niche models ----- 2

Table S1 ----- 3

Figure S1 ----- 4-8

2. Dispersal models ----- 9

Table S2 ----- 10

Figure S2 ----- 10

Figure S3 ----- 11

3. Climate change corridor (CCC) ----- 12

3.1. *maxPers* model: a single-species design framework ----- 12

3.2. *minShortfall* model: a multiple species design framework ----- 13

3.3. The search space: a constrained pool of CCCs ----- 14

Figure S4 ----- 15

Figure S5 ----- 15

4. An index to attest the representativeness of each cell within the pool of CCCs ----- 16

Figure S6 ----- 16-7

5. Spatial conflicts with socio-economic activities ----- 18

Figure S7 ----- 19-20

6. References ----- 21

## 1) Bioclimatic niche models

The study region (Iberian Peninsula and Balearic Islands, IP) was divided into 2310 10' x 10' cells, and climate suitability for each species was estimated for four periods of time (baseline, 2020, 2050, and 2080), under two IPCC-4AR greenhouse gas emission scenarios (Nakicenovic et al. 2000) (A1FI and B1). Estimates of climatic suitability through time were obtained from an ensemble of seven bioclimatic envelope models (Generalised Linear Models, Generalised Additive Models, Boosting Regression Trees, Classification Tree Analysis, Artificial Neural Networks, Mixture Discriminant Analysis and Surface Range Envelope) and three atmosphere-ocean global circulation models (CGCM2, CSIRO2, and HadCM3, for the future periods), published elsewhere (see Araújo et al. 2011). Models were fitted using a combined data set (Williams et al. 2000) of presence-absence for European mammal species (Mitchell-Jones et al. 1999). Presence-absence data was recorded in 50 km x 50 km cells and climate variables at the same resolution were obtained and averaged across 1961-1990 (baseline period). Specifically, four climatic variables were used: mean annual growing degree days (> 5°C); mean temperature of the coldest month (°C); mean total annual precipitation (mm) and a moisture index taken as the ratio of mean annual actual evapotranspiration over mean annual potential evapotranspiration (provided by the Climate Research Unit at the University of East Anglia, Mitchell et al. 2004). We then projected the bioclimatic envelope models to the IP cells using available downscaled climatic information for the baseline period and for future time periods (1990-2020 (2020), 2020-2050 (2050) and 2050-2080 (2080) averaged data) under A1FI and B1 emission scenarios (for discussion of the downscaling approach see Araújo et al. 2005). The A1FI scenario describes a globalized world under with rapid economic growth and global population that peaks in mid-century and declines thereafter and assumes rapid introduction of new and more efficient technologies. Concentrations of $CO_2$ increase from 380 ppm in 2000 to 800 ppm in 2080, and temperature rises by 3.6 K. The B1 scenario describes a convergent world with global population that peaks in mid-century and declines thereafter, as in A1, but with a rapid change toward a service and information economy and the introduction of clean and resource-efficient technology. Concentrations of $CO_2$ increase from 380 ppm in 2000 to 520 ppm in 2080, and temperature rises by 1.8 K.

Assessments of climate change impacts on biodiversity usually start with measurements of changes in the size and spatial structure of bioclimatic envelopes so to make estimates of species loss or gain (Araújo et al. 2006; Huntley et al. 2008; Peterson et al. 2002; Thomas et al. 2004; Thuiller et al. 2005). This procedure problematic if impacts of climate change are being assessed for species conservation purposes, because envelopes represent potential distributions of species and it is changes in the actual species distributions that have practical utility. Using the full bioclimatic envelopes to settle a conservation planning supporting species persistence under climate change would amount to estimating species loss from areas where they might not occur, thus undermining the usefulness of the assessments. To overcome this problem, we adopted a conservative approach for the baseline period only as future conservation options will be assessed using potential dispersal patterns for the species starting from the baseline period: Firstly, species atlas information, originally at 50km cell size, was downscaled to 10' cells such that if species

occurs in 50km cell, it was assumed to occur in each of the nested 10’ cells. Otherwise, it is predicted absent from any of the 10’ cells. Then, the baseline climatic suitability scores were filtered by the downscaled observed occurrences by setting a zero-suitability value for all cells lying outside the known distribution of species.

The suitability scores delivered by models built with atlas data (as we did) might be presumed as occurrence probabilities if we assume that sampling effort was done uniformly across all the model calibration region (Europe). The occurrence probabilities taken as that are highly dependent on the prevalence of species in the region where model was calibrated (**Table S1**). Rarer species are expected to have the lowest probability scores, even at their most suitable cells(Kadmon et al. 2003). Given that targets at the multiple-species designs were based on targets defined singularly for each of species this effect does not affect final solutions.

**Table S1** –Number of 50 km x 50 km cells in Europe and Iberia with species records in the European Atlas of Mammals.

| Species | Europe | Iberia |
|---|---|---|
| *Gpy* | 73 | 68 |
| *Ocu* | 1047 | 197 |
| *Asa* | 367 | 179 |
| *Mca* | 58 | 58 |
| *Clu* | 652 | 83 |
| *Uar* | 489 | 13 |
| *Mer* | 1426 | 55 |
| *Mlu* | 47 | 14 |
| *Fsy* | 626 | 163 |
| *Cpy* | 42 | 42 |

***Gpy**: Galemys pyrenaicus; **Mer**: Mustela erminea; **Mlu**: Mustela lutreola; **Ocu**; Oryctolagus cunniculus; **Asa**: Arvicola sapidus;*
***Mca**: Microtus cabrerae; **Clu**; Canis lupus; **Uar**. Ursus arctus; **Fsy**: Felix sylvestris; **Cpy**: Capra pyrenaica.*

We also excluded from analysis the cells showing high anthropogenic disturbance. We measured disturbance using the 1995-2004 Human Footprint Index (Sanderson et al. 2002). The index ranges from zero to 100, with the highest values representing the most impacted areas. The human footprint index is originally at 1 km x 1 km cell resolution (Wildlife Conservation Society and Center for International Earth Science Information Network 2005) and we rescaled it to 10’ cells using the averaged values. Cells with values over 60 (a value that relates with urban-like landscapes) were removed.

## *Galemys pyrenaicus*

**GRASS A1FI**

| baseline | 2020 | 2050 | 2080 |
|---|---|---|---|

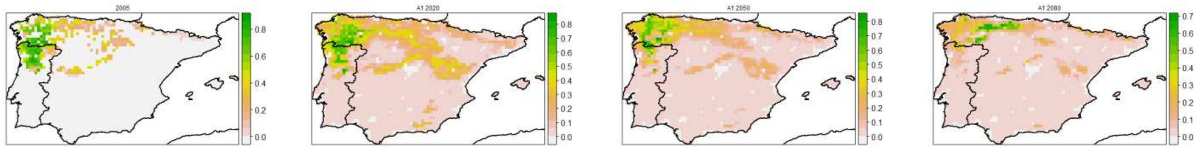

**SEDG B1**

| baseline | 2020 | 2050 | 2080 |
|---|---|---|---|

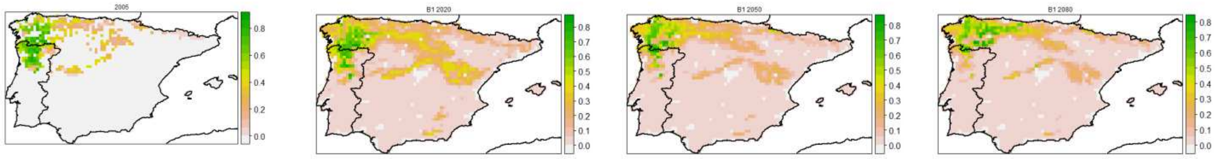

## *Mustela erminea*

**GRASS A1FI**

| baseline | 2020 | 2050 | 2080 |
|---|---|---|---|

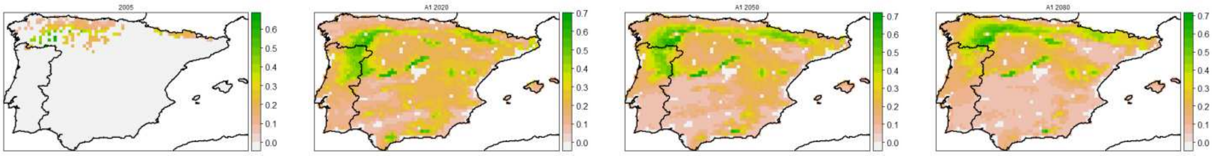

**SEDG B1**

| baseline | 2020 | 2050 | 2080 |
|---|---|---|---|

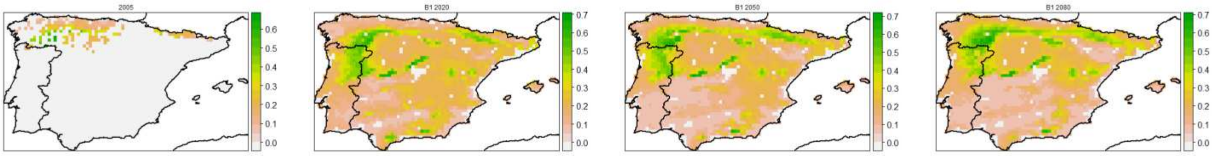

(it continues)

## *Mustela lutreola*

### GRASS A1FI

| baseline | 2020 | 2050 | 2080 |
|---|---|---|---|

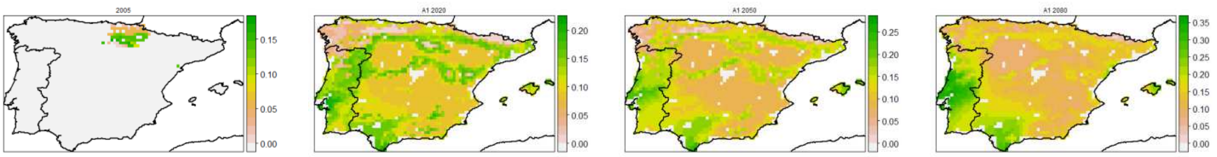

### SEDG B1

| baseline | 2020 | 2050 | 2080 |
|---|---|---|---|

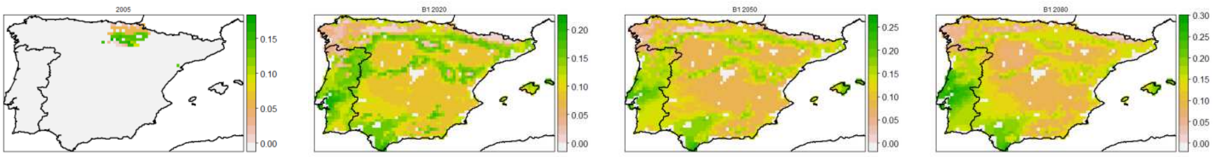

## *Oryctolagus cuniculus*

### GRASS A1FI

| baseline | 2020 | 2050 | 2080 |
|---|---|---|---|

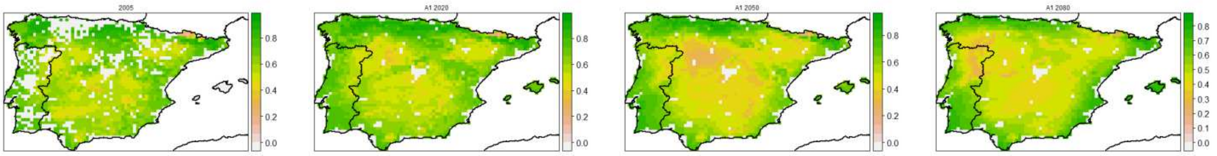

### SEDG B1

| baseline | 2020 | 2050 | 2080 |
|---|---|---|---|

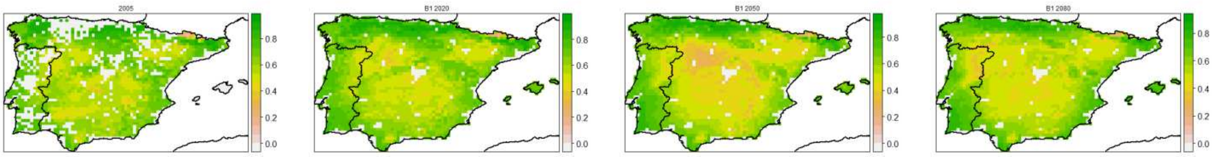

(it continues)

## *Arvicola sapidus*

### GRASS A1FI

**baseline** **2020** **2050** **2080**

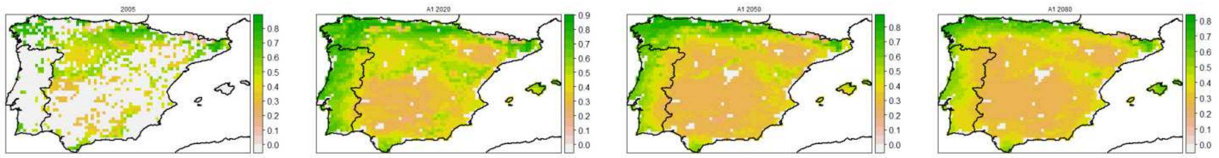

### SEDG B1

**baseline** **2020** **2050** **2080**

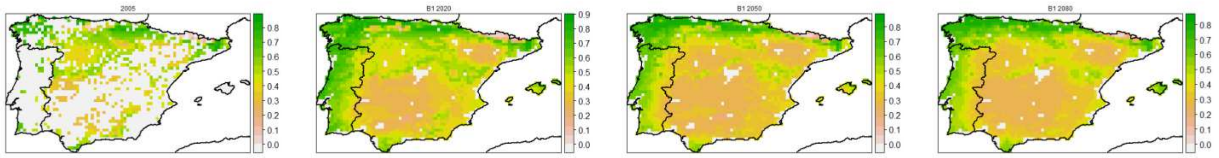

## *Microtus cabrerae*

### GRASS A1FI

**baseline** **2020** **2050** **2080**

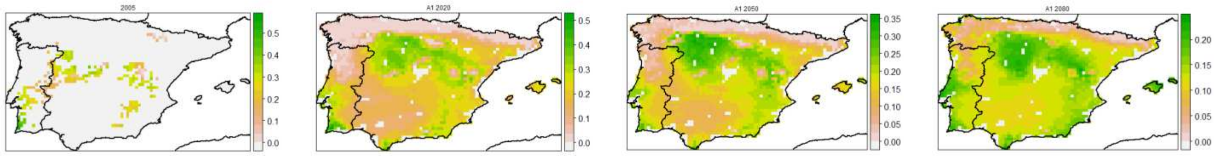

### SEDG B1

**baseline** **2020** **2050** **2080**

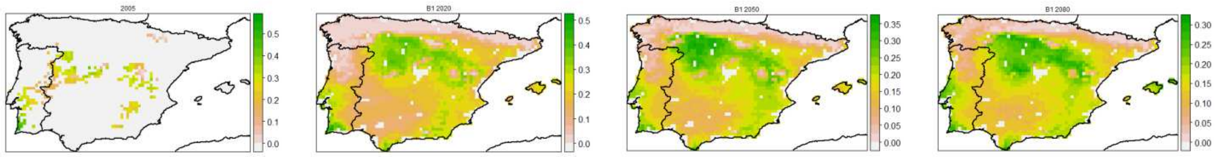

(it continues)

## *Canis lupus*

### GRASS A1FI

| baseline | 2020 | 2050 | 2080 |
|---|---|---|---|

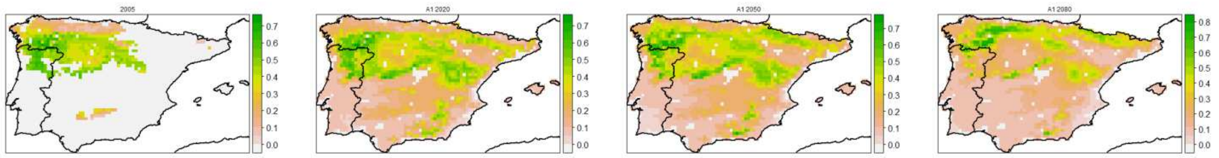

### SEDG B1

| baseline | 2020 | 2050 | 2080 |
|---|---|---|---|

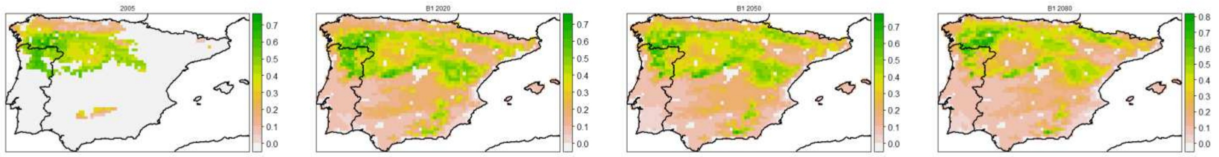

## *Ursus arctus*

### GRASS A1FI

| baseline | 2020 | 2050 | 2080 |
|---|---|---|---|

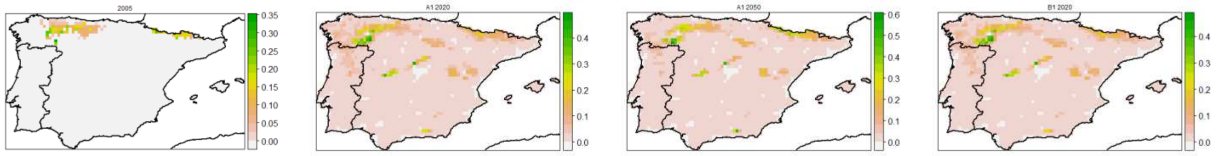

### SEDG B1

| baseline | 2020 | 2050 | 2080 |
|---|---|---|---|

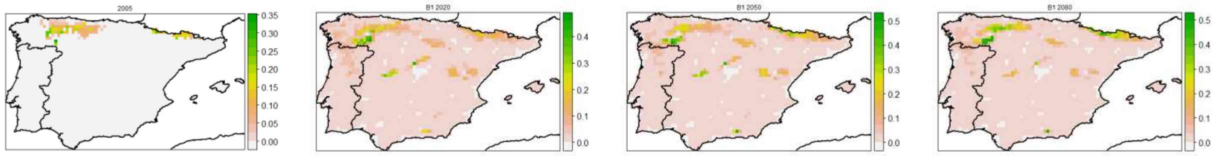

(it continues)

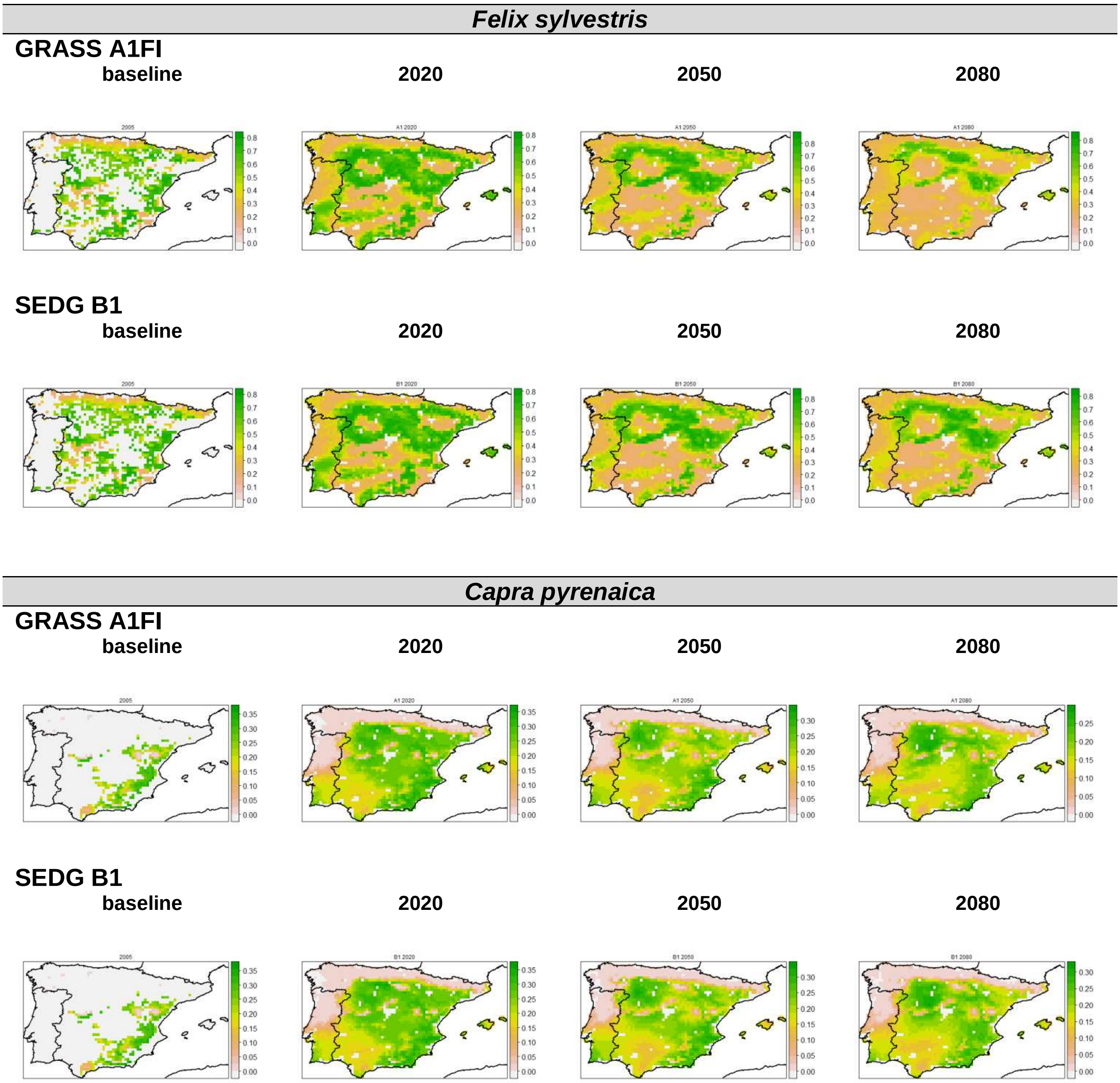


**Figure S1 –** Climatic suitability predictions for ten non-volant mammal species considered threatened in Iberian Peninsula and Canary Islands for baseline, 2020, 2050 and 2080 time periods under two socio-economic storylines (GRASS – A1FI and SEGD – B1).

## 2) Dispersal models

We used the framework published by Schloss *et al.*(2012) to estimate the maximum distance that each species is able to disperse in a 30 year time period (*Dmax*) (the time-interval assessed). In this framework dispersal ability is taken as a function of body weight (*w*, in kg) and diet type (herbivorous and carnivores) that define distance covered by single-natal events (*Dnatal*) and generation time (*gt*, in years) that translates these distances into a time frame. In their most permissive estimates for dispersal abilities in a horizon of time of 110 years:

$$Dnatal = 3.45 \times w^{0.89} \text{ (for carnivores)}$$

$$Dnatal = 1.45 \times w^{0.54} \text{ (for herbivorous)}$$

$$Dmax = Dnatal \times gt$$

We took body weight (average from the minimum and maximum reported values), generation time and diet type data from the IUCN Red List of Portugal (Cabral et al. 2005) and Spain (Dirección general para la biodiversidad 2007) (**Table S1**).

The estimated *Dmax* values for *Canis lupus* and *Ursus arctus* were substantially large (1234 and 2857 km, respectively) considering the surface area of IP and the likelihood of barrier effects in a region characterized by high human population density. We restricted *Dmax* for the two species to 200 km (the estimated *Dmax* for the most similar among the other analyzed species) (**Table S2**).

We considered negative exponential kernel curves to reflect the probability of each species to disperse successful across space, $pd_s^{u1,u2,}$ depending on the Euclidean distance (*dist*) between a source (u1) and a terminal point (u2):

$$pd_s^{u1,u2} = \begin{cases} \exp(-\alpha.\dfrac{dist(u1,u2)}{Dmax}, & \text{if } dist(u1,u2) \leq Dmax \\ 0 & \text{,otherwise} \end{cases}$$

For all species, we assumed three curves reflecting 15, 10 and 5% of successful dispersal at the maximum dispersal distances (**Fig. A1, Fig. A2**), such that:

$$dist(u1,u2) = Dmax \Rightarrow \begin{cases} pd_s^{u1,u2} = 0.15 \quad \Rightarrow \quad \alpha = \alpha 1 = 1.887 \\ pd_s^{u1,u2} = 0.10 \quad \Rightarrow \quad \alpha = \alpha 2 = 2.303 \\ pd_s^{u1,u2} = 0.05 \quad \Rightarrow \quad \alpha = \alpha 3 = 2.996 \end{cases}$$

Given that the landscapes tend to be heterogeneous and perceived differently by distinct species this is likely to be a simplistic assumption. However data deficiencies and analytical complexities, computing functional distances for each species (Aben et al. 2016) would be largely demanding and outside the main scope of this study. Given the large area covered by each cell (see next section) we considered that every cell might potentially be used by all the species to disperse and, therefore, a geographic distance might be an acceptable proxy to represent dispersal processes.

**Table S2**–Data used to model maximum dispersal distance (*Dmax*) in a 30-years time period.

| Species | Diet type | Body weight (kg) | | Generation time (yr) | *Dnatal* (km) | *Dmax* (km) | |
|---|---|---|---|---|---|---|---|
| | | Min. | Max. | | | Estim. | Final |
| *Arvicola sapidus* | Herbivore | 0.140 | 0.330 | 1.00 | 0.66 | 19.90 | 20.00 |
| *Galemys pyrenaicus* | Herbivore | 0.050 | 0.076 | 1.00 | 0.33 | 9.78 | 10.00 |
| *Microtus cabrerae* | Herbivore | 0.040 | 0.075 | 1.00 | 0.31 | 9.30 | 10.00 |
| *Oryctolagus cuniculus* | Herbivore | 0.970 | 1.460 | 0.75 | 1.61 | 64.43 | 65.00 |
| *Mustela erminea* | Carnivore | 0.180 | 0.330 | 1.00 | 1.02 | 30.67 | 31.00 |
| *Mustela lutreola* | Carnivore | 0.700 | 1.100 | 0.83 | 3.14 | 113.08 | 115.00 |
| *Caprea pyrenaica* | Herbivore | 31.000 | 90.000 | 2.50 | 13.29 | 159.48 | 160.00 |
| *Felix sylvestris* | Carnivore | 3.000 | 6.400 | 2.00 | 13.68 | 205.15 | 200.00 |
| *Canis lupus* | Carnivore | 27.000 | 32.000 | 1.83 | 75.41 | 1147.00 | 200.00 |
| *Ursus arctus* | Carnivore | 90.000 | 250.000 | 3.50 | 333.36 | 2857.41 | 200.00 |

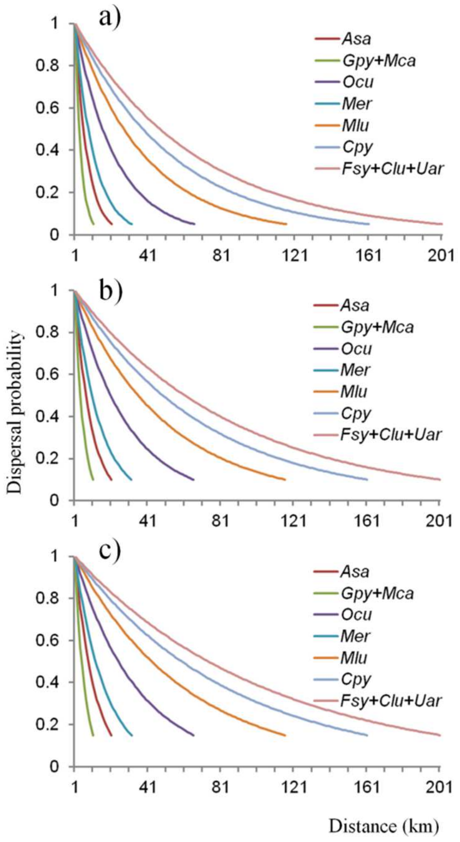


**Figure S2** - The kernel curves used to model dispersal ability of each analysed species in a 30 year time period as a function of the Euclidean distance between a source and a terminal cell. We settled three scenarios varying the probability of successful dispersal at the maximum dispersal distance: a) $\alpha_1$: 0.05; b) $\alpha_2$: 0.10, and c)$\alpha_3$: 0.15. *Asa - Arvicola sapidus; Gpy -Galemys pyrenaicus; Mca - Microtus cabrerae; Ocu - Oryctolagus cuniculus;Mer - Mustela erminea; Mlu - Mustela lutreola; Cpy - Caprea pyrenaica; Fsy - Felix sylvestris; Clu - Canis lupus; Uar - Ursus arctus.*

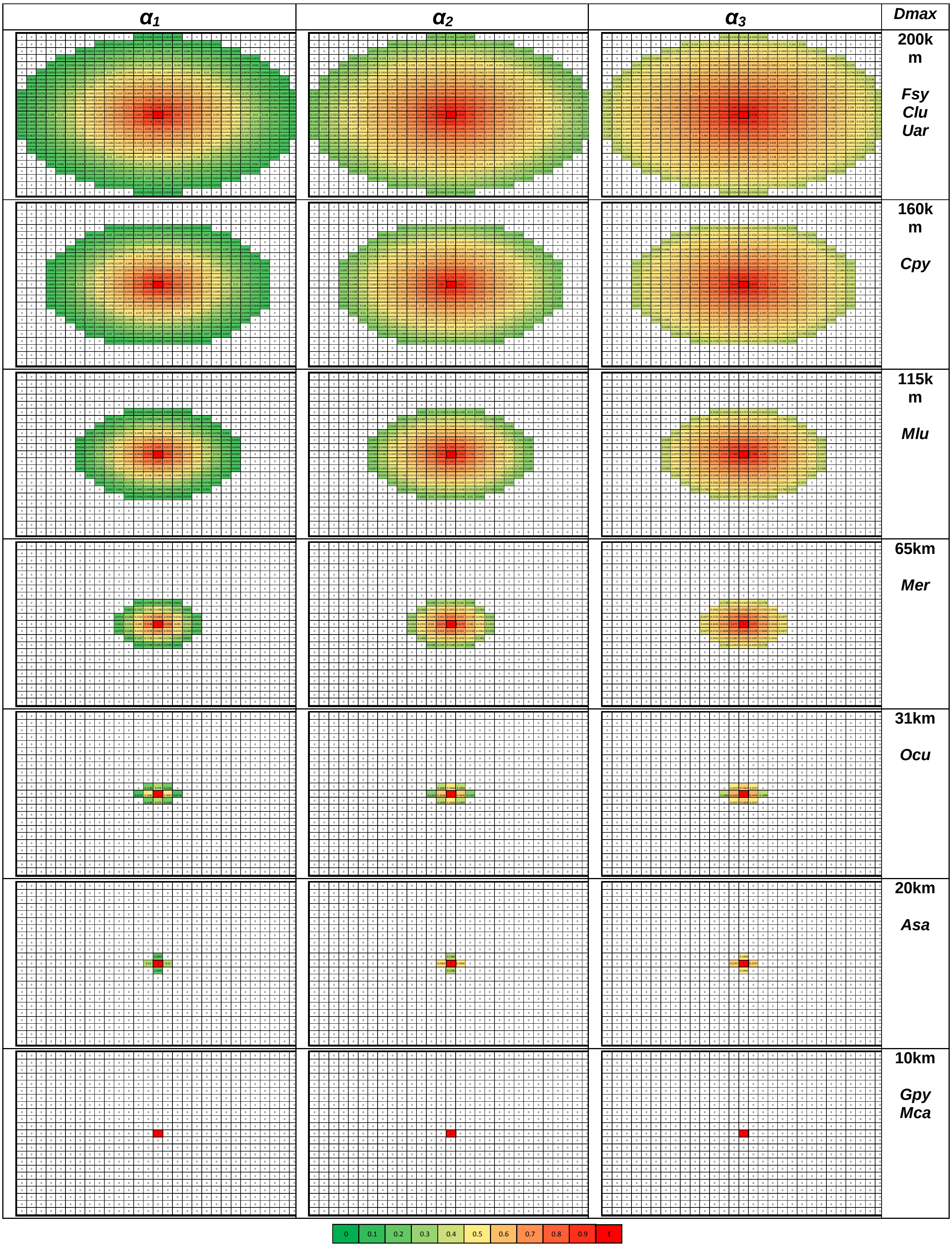


**Figure S3** – Mapping ofspecies' dispersal kernel curves for a 30 year time period in 10' grid-cells at the latitude of Iberia.

*Asa - Arvicola sapidus; Gpy -Galemys pyrenaicus; Mca - Microtus cabrerae; Ocu - Oryctolagus cuniculus;Mer - Mustela erminea; Mlu - Mustela lutreola; Cpy - Caprea pyrenaica; Fsy - Felix sylvestris; Clu - Canis lupus; Uar - Ursus arctus.*

### 3) Climate change corridor

In our analysis we used the concept of climate change corridors, CCCs (sensu Alagadoret al. 2014, originally introduced by Williams et al. 2005 and improved by Phillips et al. 2008) to optimize conservation decisions under a climate change context. A CCC defines a sequence of time-ordered planning units to be occupied by the species with time (given species-specific climatic tolerances and dispersal constraints). The probability of a species *s* to persist in a given corridor *c*, formed by planning units *u1, u2, ..., uT*, selected for time-periods *t* =1, 2,...,*T* is settled as:

$$P_s^c = po_s^{u1} \times pd_s^{u1,u2} \times po_s^{u2} \times \ldots \times po_s^{u(T-1)} \times pd_s^{u(T-1),uT} \times po_s^{uT} \qquad \text{(S1)}$$

Where $po_s^{ut}$ is the probability of species *s* to occur in planning unit *ut* at time *t*, and $pd_s^{u(t-1),ut}$ is the probability of species *s* to successfully disperse between *u(t-1)* and *ut* in the time-interval [*t*-1,*t*] (taken from the dispersal kernel). The persistence of a species *s* in a set *C* of CCCs is the maximum sum of persistence scores in a set of independent corridors of *C*, and a set of corridors is independent if no two corridors intersect (i.e. use the same planning unit at the same time–period). The concept of independent corridors was introduced by Williams et al. 2005 as a convenient property to mitigate the spread of unforeseen negative contagious events. Hence, when calculating the persistence of a species along a set *C* of CCCs we only account for independent corridors in *C*.

The CCCs might serve distinct conservation requisites (Alagador et al. 2016) and define conservation units carrying a spatial (where to prioritize) and a temporal dimension (when to do so) for planners to evaluate.

#### 3.1) *maxPers* model: a single-species design framework

This model permits to obtain for each species the maximum possible persistence in independent CCCs covering a maximum established budgetary/areal constraint. Therefore we ran that problem to emulate single-species (SS) planning designs, where *[S]¹={{s}: s ∈S}* is a variable set of size one containing each individual species. The model is formulated as:

$$\begin{cases} \max \sum_{c \epsilon cor_s} P_s^c . z_s^c & \text{(S2)} \\ s.t. \begin{vmatrix} \sum_{c \epsilon cor_s^{u,t}} z_s^c \leq 1 \quad ,\forall s \epsilon [S]^1, \forall u \epsilon U, t = 1, \ldots, T & \text{(S3)} \\ x_u^t \geq z_s^c \quad ,\forall s \epsilon [S]^1, \forall u \epsilon U, \forall c \epsilon cor_s^{u,t}, t = 1, \ldots, T & \text{(S4)} \\ \sum_{u \epsilon U} \sum_{t \epsilon T} c_u^t . x_u^t \leq B & \text{(S5)} \\ x_u^t \epsilon [0,1] \quad ,\forall u \epsilon U, t = 1, \ldots, T & \text{(S6)} \\ z_s^c \epsilon \{0,1\} \quad ,\forall s \epsilon [S]^1, \forall c \epsilon cor_s & \text{(S7)} \end{vmatrix} \end{cases}$$

In this formulation variables $z_s^c$ inform on the selection of corridor *c* ($z_s^c = 1$ if selected; $z_s^c = 0$ if not selected). The set $cor_s^{u,t}$ defines a set of corridors potentially available for species *s* converging in cell *u* at time period *t*. Therefore, the

constraints, **Equations (S3),** limit the selection of a corridor within that set to one, in order to guarantee that the CCCs selected are functionally independent (thus avoiding propagation of deleterious contagious processes). The constraints, **Equations (S4),** associate each variable related with cell selection to the respective corridors where they occur in a given time period, $cor_s^{u,t}$ such that $x_u^t$=1 when the corridor *c* where cell *u* is selected in time *t* for species *s* is selected ($z_s^t$=1) and $x_u^t$=0, otherwise. Constraint, **Equation (S5)** records the budgetary/areal limitation, with *B* defining the budget available (conflict allowed) and $c_u^t$ the cost/conflict associated to the selection of cell *u* in time *t*. The remaining constraints, **Equations (S6-S7**) establish the values able to be taken by each variable. The function at **Equation (S2)** embodies the driving objective of the model (i.e.to maximize the persistence for a within the area allocated through time, (i.e. CCCs).

### 3.2) *minShortfall* model: a multiple species design framework

In this model we developed a CCC-model with the objective of minimizing the summed shortfalls ($\Delta_s$) between predefined species persistence targets ($P_s$) and the ones that are accomplished under a budgetary constraint that limits the selection of corridor areas (*B*), **Equation (S8**). Although persistence targets may be defined to guarantee an adequate status of species protection, they do not reflect an intuitive control of the result as it reflects a probability of many factors to be successful. We therefore establish as a persistence target for a species, the persistence obtained in the areas identified in the *maxPers* model under the same budgetary constrain, *B*.

$$\begin{cases} \min \sum_{s\epsilon S} \Delta_s & \text{(S8)} \\ s.t. \left| \begin{array}{ll} \Delta_s \geq P_s - \frac{(\sum_{c\epsilon cor_s} P_s^c . z_s^c)}{P_s}, \forall s\epsilon S & \text{(S9)} \\ \sum_{c\epsilon cor_s^{u,t}} z_s^c \leq 1 \quad , \forall s\epsilon S, \forall u\epsilon U, t=1,\ldots,T & \text{(S10)} \\ x_u^t \geq z_s^c \quad , \forall s\epsilon S, \forall u\epsilon U, \forall c\epsilon cor_s^{u,t}, t=1,\ldots,T & \text{(S11)} \\ \sum_{u\epsilon U} \sum_{t\epsilon T} c_u^t . x_u^t \leq B & \text{(S12)} \\ x_u^t \epsilon [0,1] \quad , \forall u\epsilon U, t=1,\ldots,T & \text{(S13)} \\ z_s^c \epsilon \{0,1\} \quad , \forall s\epsilon S, \forall c\epsilon cor_s & \text{(S14)} \\ \Delta_s \geq 0 \quad , \forall s\epsilon S & \text{(S15)} \end{array} \right. \end{cases}$$

For each species constraints, **Equations (S9**), record the shortfall between the predefined persistence target and the one obtained by summing the species persistence in each of the selected corridors ($P_s^c$). The constrains, **Equations (S10-S14),** are similar to the ones in *maxPers*. Constraints in **Equations (S15)** establish the domain of $\Delta_s$ variables.

### 3.3) The search space: a constrained pool of CCCs

Because solving both problems to optimality is an hard task (i.e., large time consumption and computational resources) for case studies involving a large number of cells, time periods and/or species, an informed reduction of the search space shows practical utility with reduction of solving times, although at the expense of solution optimality (for a discussion on the optimal vs heuristic debate in conservation planning see, Moilanen 2008). We met this need within our case study and therefore we developed an approach that reduces the number of CCCs to be evaluated to the ones showing the highest persistence expectancies for each of the analyzed species. To manage this goal we converted distributional data for each species along time into a network flow (Ahuja et al. 1993), directed, multipartite graph representation, with nodes representing cells with non-null climatic suitability. Each time period is represented by a pair of node-partitions and by arcs linking sibling nodes (i.e., related with the same cell). Each of these arcs carry a cost associated with the species occurrence probability in the respecting cell *ut* (cost=$-log(po_s^{ut})$). The other type of arcs links nodes in the second partition in time *t* to the nodes in the first partition in time *t+1* that are potentially reachable by the species through dispersal. These arcs carry a cost related with the probability of successful dispersal between *ut* and *u(t+1)* (cost=$-log(pd_s^{ut,u(t+1)})$). A source node where arcs are originated to all the nodes in the first partition (cost=0) and a terminal node receiving arcs from all the nodes in the final partition (cost=0) are also delineated. In such a graph a path refers to a set of edges that link the source to the terminal node (**Fig. A4**) and the cost of such a path ($cost^{PATH}$) is obtained by summing the costs of edges composing it. A climate change corridor equates therefore to such a path and it is composed by the cells (in the respective time periods) involved in selected edges. The expected persistence of the species in this corridor ($P_s^c$=$-exp(cost^{PATH})$).

We implemented the algorithm developed by Martins *et al.*(1999) to select for each species the *k* least cost paths. These paths correspond to the *k* climate change corridors with the highest persistence values ($P_s^c$) (**Fig. A5**). The final solutions *maxPers* and *minShortfall* were obtained restricting CCCs selections to the top-persistence corridor pool obtained for each climate and dispersal scenario. We used the commercial mixed integer programming solver IBM ILOG CPLEX version 12 (ILOG 2011) to run problems to optimality (sub-optimality branch and bound gap < 0.01).

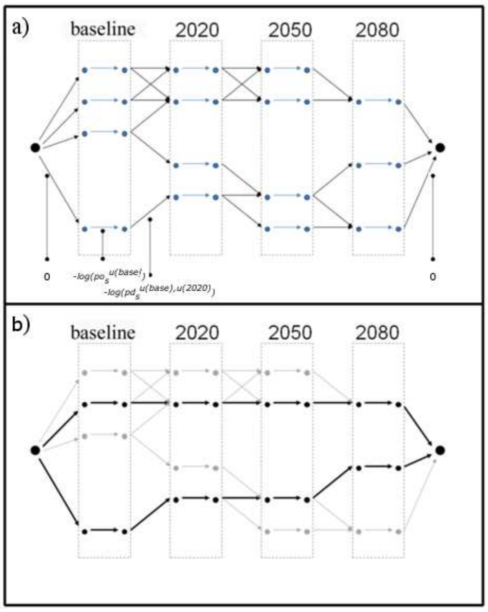


**Figure S4 –** An illustrative graph representation of the required data used for the identification of the top persistence CCC for one species from a baseline period up to 2080. a) Nodes represent cells with non-null climatic suitability for the species in different periods. Edges within each period link nodes corresponding to the same cell and have a cost of $-log(po_s^{ut})$. Edges in-between consecutive periods link cells distancing less the maximum dispersal capacity of the species and have a cost of $-log(pd_s^{ut,u(t+1)})$. Edges from and to the source and terminal nodes, respectively, have a zero-cost. b) An example of two climate change corridors (bold lines) (i.e., paths linking the source and terminal nodes).

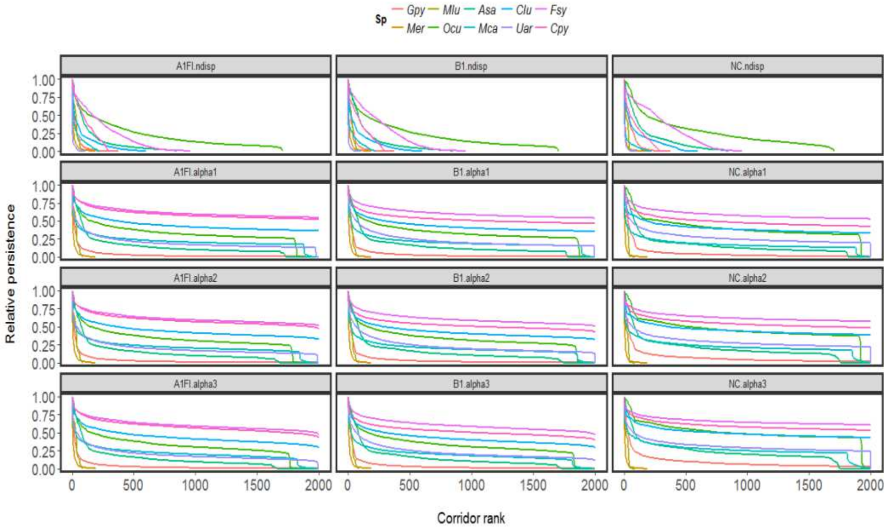


**Figure S5 –** Ordered distribution of species persistence scores (proportion of maximum values) associated with the pooled CCCs. Scenarios span distinct climate change storylines (A1FI, B1 and NC: no-change) and species dispersal abilities (ndisp: no-dispersal, α1, α2 and α3). ***Gpy****: Galemys pyrenaicus;* ***Mer****: Mustela erminea;* ***Mlu****: Mustela lutreola;* ***Ocu****; Oryctolagus cunniculus;* ***Asa****: Arvicola sapidus;* ***Mca****: Microtus cabrerae;* ***Clu****; Canis lupus;* ***Uar****. Ursus arctus;* ***Fsy****: Felix sylvestris;* ***Cpy****: Capra pyrenaica.*

## 4) An index to attest the representativeness of each cell within the pool of CCCs

Given that the final solutions delivered by CCC-models are quite dependent on complex relationships between i) cell costs; ii) the changing patterns of climatic suitability for the multiple targeted species, and; iii) the species dispersal abilities (and respective successful colonization). Thus it might be relevant to consider at a previous point in the analyses the representativeness of each cell, *u*, within the pool of CCCs for a set of species *S*, as a measure of the likelihood of each cell to be part of final solutions (**Fig. S6**):

$$R_u = \sum_s^S R_u^s \qquad (S15)$$

where, for each species, *s*:

$$R_u^s = \frac{1}{R_{ALL}^s} \sum_c^{cor_s} \sum_u^{U(c)} \sum_t^T P_s^c . 1(t), \text{ where } 1(t): \begin{cases} 1 & \text{if } u \text{ defines time-period } t \text{ in corridor } c \\ 0 & \text{otherwise} \end{cases} \qquad (S16)$$

where *U(c)* is the set of cells representing the corridor *c* among all the *T* time-periods under analysis, and $R_{ALL}^s$ is the representativeness associated to all the cells present in all the CCCs in the pool for species *s*:

$$R_{ALL}^s = T \times \sum_c^{cor_s} P_s^c \qquad (S17)$$

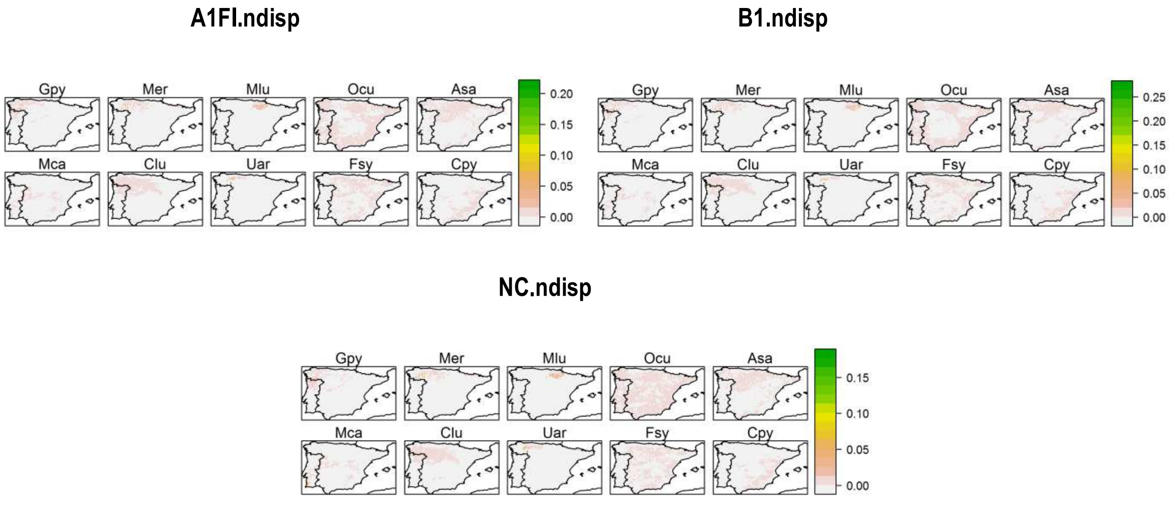


(it continues)

**A1FI.α1**

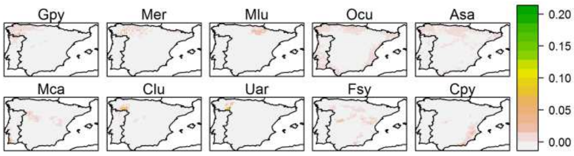


**B1. α1**

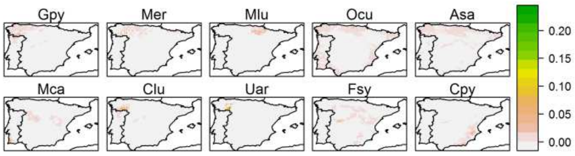


**NC. α1**

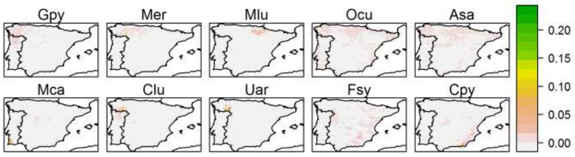


**A1FI.α2**

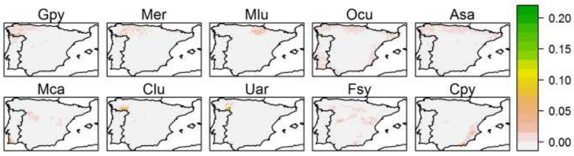


**B1. α2**

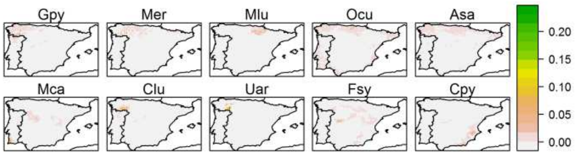


**NC. α2**

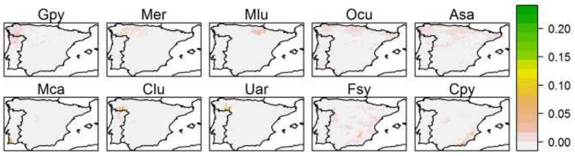


**A1FI.α3**

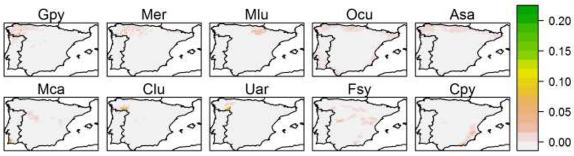


**B1. α3**

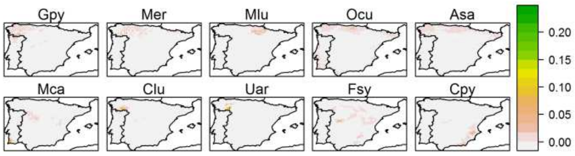


**NC. α3**

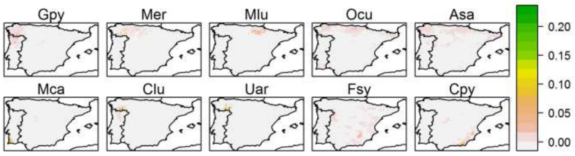


**Figure S6** – Representativeness of cells in the CCCs pools for the ten species under analysis. Scenarios span the A1FI, B1 and NC no-change climate storylines and the ndisp: non-dispersal; α1, α2 and α3 dispersal parameterizations.***Gpy****: Galemys pyrenaicus;* ***Mer****: Mustela erminea;* ***Mlu****: Mustela lutreola;* ***Ocu****; Oryctolagus cunniculus;* ***Asa****: Arvicola sapidus;* ***Mca****: Microtus cabrerae;* ***Clu****; Canis lupus;* ***Uar****. Ursus arctus;* ***Fsy****: Felix sylvestris;* ***Cpy****: Capra pyrenaica.*

### 5) Spatial conflicts with socio-economic activities

We analyzed the impact of different socio-economic activities over the effectiveness of CCCs in facilitating species persistence. We used land-cover data to simulate the level of cell-occupancy by the most relevant socio-economic activities in the region. We classified those activities is broad classes: agriculture, cattle raising/ pastures (hereon named by pastures), forestry and urban activities (e.g. industry and commerce) (Jantz et al. 2015). For the baseline period land-cover was obtained through the CORINE Land Cover 2006 website (http://www.eea.europa.eu/data-and-maps/data/corine-land-cover-2006-raster) at 250m resolution using the level-I classification (i.e. built-up areas, arable lands, permanent crops, grasslands, forest and others). Future land cover at the same resolution was derived from the EU projects ALARM and ECOCHANGE (Dendoncker et al. 2006; Rounsevell et al. 2006)that spanned the period 2006-2080. To match climatic data, we retained land-cover data for 2021-2050 and 2051-2080. We used two socio-economic storylines developed under ALARM that are consistent with the AR4 climate change scenarios (growth and applied strategy, GRAS, considered equivalent to A1FI, and sustainable European development goal, SEDG, considered equivalent to B1). We rescaled land cover data into the 10´-resolution grid by recording the fraction covered by each land-cover class in each cell (i.e. occupancy) (**Fig. S7**). In the assessments that assume a stable NC climate we replicated for the future time-periods the land-use patterns estimated for the current time-period.

We depicted an index to characterize the levels of conflict between conservation actions and each socio-economic activity in each cell ($confl_i$).

$$confl_i = confl_{AREA} + confl_i^{se} \qquad \text{(S18)}$$

Given that all cells present the same area $confl_i$ =1 and the variation of $confl_i$ expresses variations on the occupancy of land by the socio-economic activities ($confl_i^{se}$). We evaluated distinct socio-economic layers: 1) uniform –this is a simulated layer assuming no occupancy of socio-economic activities (i.e. wilderness scenario) along all the Iberia, such that $confl_i$ = 1 for all cells; 2) agriculture; 3) forestry; 4) pasture, in which $confl_i^{se}$ records the occupancy of each socio-economic activity, respectively; 5) urban – assuming that industrial and commercial activities return the highest revenues when compared with revenues from primary-sector activities, $confl_i^{urban}$ was settled as twice the estimated urban occupancy in *i*, and; 6) total – in which $confl_i^{se} = \Sigma_{se}\ confl_i^{se}$.with *se* representing agriculture, forestry, pasture and urban activities.

We carried out analyses using a sequence of 400 allowed-conflict values, starting at four conflict-units (i.e. the minimum value to define a single corridor for four time-periods, that is taken from the homogeneous, uniform conflict scenario), $B$ = 4, 8, 12, …, 1600. In the assessments that assume a stable NC climate we replicated for the future time-periods the land-use patterns estimated for the baseline period.

a) Agriculture

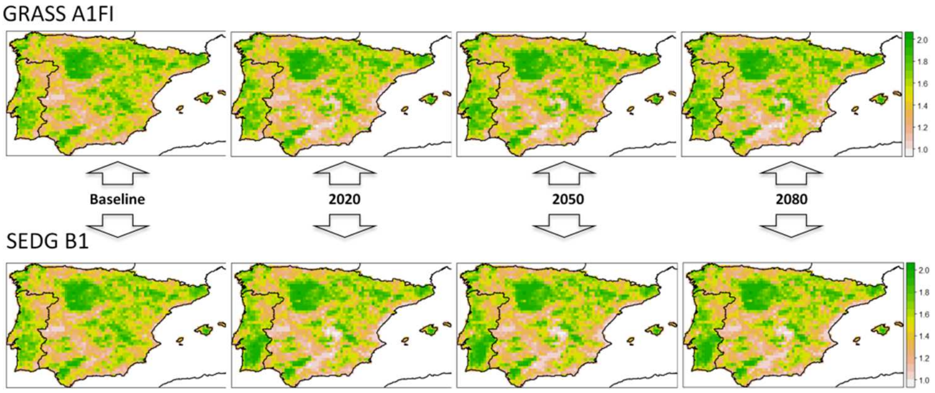


b) Pasture lands

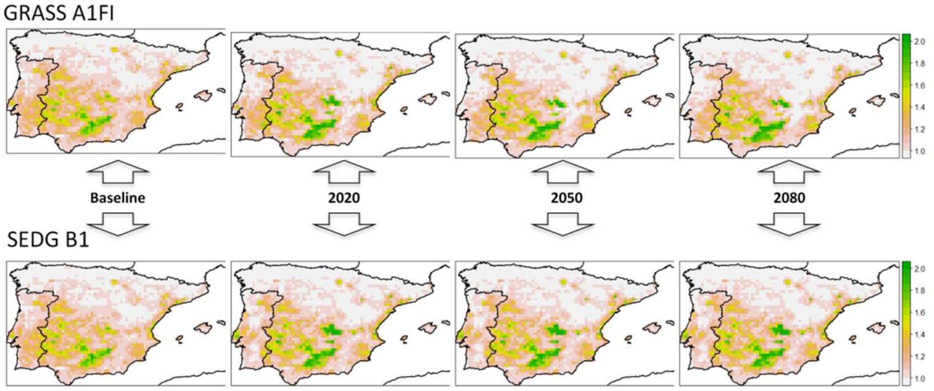


c) Forestry

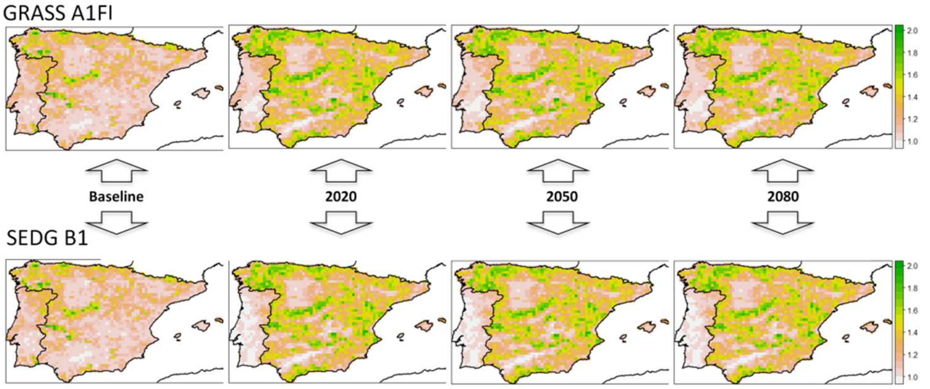


(it continues)

d) Urban areas

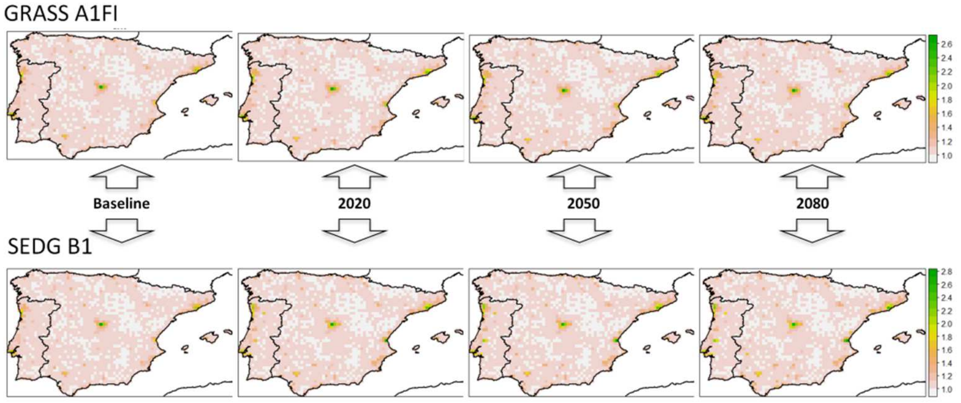


e) All conflicts

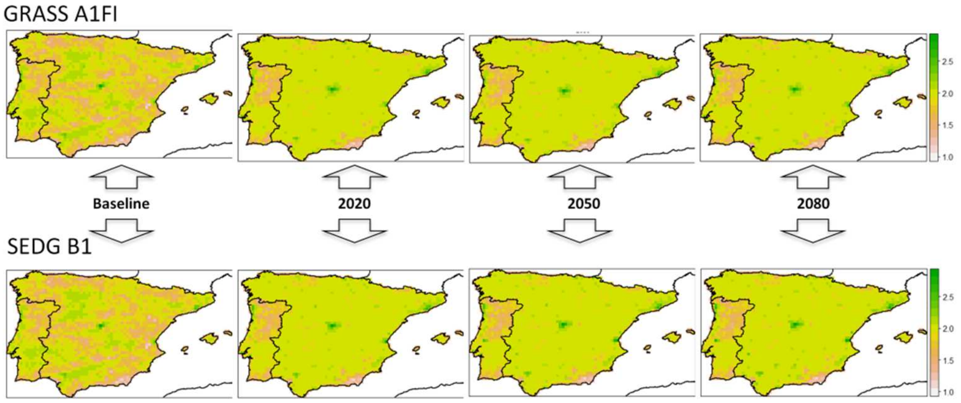


**Figure S7 – Spatial conflicts of conservation areas and the major socio-economic activities** in Iberia from a baseline period into 2080 under two changing scenarios (GRASS - A1FI and SEDG – B1). The analyses were performed for **a)** agriculture; **b)** cattle-raising/pastures; **c)** forestry; **d)** urban areas and **e)** all the previous ones together (total).

## 6) References in Appendix 1


Aben J., Bocedi G., Palmer S.C.F. *et al.* (2016) The importance of realistic dispersal models in conservation planning: application of a novel modelling platform to evaluate management scenarios in an Afrotropical biodiversity hotspot. *Journal of Applied Ecology* **53**, 1055-1065.

Ahuja R.K., Magnanti T.L., Orlin J.B. (1993) *Network Flows: Theory, Algorithms and Applications*. Prentice Hall, Upper Saddle River, New Jersey, USA.

Alagador D., Cerdeira J.O., Araújo M.B. (2014) Shifting protected areas: scheduling spatial priorities under climate change. *Journal of Applied Ecology* **51**, 703-713.

Alagador D., Cerdeira J.O., Araújo M.B. (2016) Climate change, species range shifts and dispersal corridors: an evaluation of spatial conservation models. *Methods in Ecology and Evolution* **7**, 853-866.

Araújo M.B., Alagador D., Cabeza M., Nogués-Bravo D., Thuiller W. (2011) Climate change threatens European conservation areas. *Ecology Letters* **14**, 484-492.

Araújo M.B., Thuiller W., Pearson R.G. (2006) Climate warming and the decline of amphibians and reptiles in Europe. *Journal of Biogeography* **33**, 1712-1728.

Araújo M.B., Thuiller W., Williams P.H., Reginster I. (2005) Downscaling European species atlas distributions to a finer resolution: implications for conservation planning. *Global Ecology and Biogeography* **14**, 17-30.

Cabral M.J., Almeida J., Almeida P.R. *et al.* (2005) *Livro Vermelho dos Vertebrados de Portugal. Instituto da Conservação da Natureza. Lisboa. 659p.* Instituto da Conservação da Natureza, Lisboa, Portugal.

Dendoncker N., Bogaert P., Rounsevell M. (2006) A statistical method to downscale aggregated land use data and scenarios. *Journal of Land Use Science* **1**, 63-82.

Dirección general para la biodiversidad. (2007) *Atlas y libro rojo de los mamíferos terrestres de España*. Organismo Autónomo Parques Nacionales, Madrid España.

Huntley B., Collingham Y.C., Willis S.G., Green R.E. (2008) Potential Impacts of Climatic Change on European Breeding Birds. *PLoS ONE* **3**, e1439.

ILOG. (2011) *CPLEX 12.3 Reference Manual.*

Jantz S.M., Barker B., Brooks T.M. *et al.* (2015) Future habitat loss and extinctions driven by land-use change in biodiversity hotspots under four scenarios of climate-change mitigation. *Conservation Biology* **29**, 1122-1131.

Kadmon R., Farber O., Danin A. (2003) A systematic analysis of factors affecting the performance of climatic envelope models. *Ecological Applications* **13**, 853-867.

Martins E.d.Q.V., Pascoal M.M.B., Santos J.L.E.d. (1999) Deviation algorithms for ranking shortest paths. *International Journal of Foundations of Computer Science* **10**, 247-261.

Mitchell-Jones A.J., Amori G., Bogdanowicz W. *et al.* (1999) *The Atlas of European Mammals*. Academic Press, London.

Mitchell T., Carter T.R., Jones, P.,, Hulme M. (2004) A comprehensive set of high-resolution grids of monthly climate for Europe and the globe: the observed record (1901-2000) and 16 scenarios (2001-2100). *Tyndall Centre Working Papers.* Tyndall Centre, Norwich.

Moilanen A. (2008) Two paths to a suboptimal solution – once more about optimality in reserve selection. *Biological Conservation* **141**, 1919-1923.

Nakicenovic N., Alcamo J., Davis G. *et al.* (2000) *Special Report on Emissions Scenarios: A Special Report of Working Group III of the Intergovernmental Panel on Climate Change*. Cambridge University Press, Cambridge, U.K.

Peterson A.T., Ortega-Huerta M.A., Bartley J. *et al.* (2002) Future projections for Mexican faunas under global climate change scenarios. *Nature* **416**, 626-629.

Phillips S., Williams P., Midgley G., Aaron A. (2008) Optimizing dispersal corridors for the Cape Proteaceae using network flow. *Ecological Applications* **18**, 1200-1211.

Rounsevell M.D.A., Reginster I., Araújo M.B. *et al.* (2006) A coherent set of future land use change scenarios for Europe. *Agriculture, Ecosystems & Environment* **114**, 57-68.

Sanderson E.W., Jaiteh M., Levy M.A., Redford K.H., Wannebo A.V., Woolmer G. (2002) The Human Footprint and the Last of the Wild. *BioScience* **52**, 891-904.

Schloss C.A., Nuñez T.A., Lawler J.J. (2012) Dispersal will limit ability of mammals to track climate change in the Western Hemisphere. *Proceedings of the National Academy of Sciences* **109**, 8606–8611.

Thomas C.D., Cameron A., Green R.E. *et al.* (2004) Extinction risk from climate change. *Nature* **427**, 145-148.

Thuiller W., Lavorel S., Araújo M.B., Sykes M.T., Prentice I.C. (2005) Climate change threats plant diversity in Europe. *Proceedings of the National Academy of Sciences, USA* **102**, 8245-8250.

Wildlife Conservation Society W.C.S., Center for International Earth Science Information Network C.C.U. (2005) Last of the Wild Project, Version 2, 2005 (LWP-2): Global Human Footprint Dataset (IGHP). NASA Socioeconomic Data and Applications Center (SEDAC), Palisades, NY.

Williams P., Hannah L., Andelman S. *et al.* (2005) Planning for climate change: Identifying minimum-dispersal corridors for the Cape Proteaceae. *Conservation Biology* **19**, 1063-1074.

Williams P., Humphries C., Araújo MB *et al.* (2000) Endemism and important areas for representing European biodiversity: a preliminary exploration of atlas data for plants and terrestrial vertebrates. *Belgian Journal of Entomology* **2**, 21-46.
