# Supplementary material for: A quantitative analysis on the effects of critical factors limiting the effectiveness of species conservation in future time
Source: Ecol Evol. 2018 Feb 24;8(6):3457–67. doi: 10.1002/ece3.3788 (PMC5869367; doi:10.1002/ece3.3788)
Supplement: Supplementary file 2 [file ECE3-8-3457-s002.pdf]

SUPPLEMENTARY MATERIAL

APPENDIX 2

- FIGURES -

CONTENTS

1. Effect size analysis ----- 2

  Figure S8 ----- 3-5

  Figure S9 ----- 6-8

  Figure S10 ----- 9-10

2. References ----- 11

  Figure S11 ----- 12-15

  Figure S12a ----- 16

  Figure S12b ----- 17

  Figure S13 ----- 18

  Figure S14 ----- 19

1) Effect size analysis

We conducted linear mixed model analyses to estimate the effect sizes of planning design, climate, dispersal and conflict type on the persistence of each species in the CCCs, using the 400 assessed conflict levels as a random factor and assuming only additive fixed-factorial terms (i.e., no interactions) (**see Appendix-S2 for details**). Analyses were performed after testing for homoscedasticity (Levene's tests) and normality (Shapiro Wilk tests), which are required to inference accuracy in linear models Model accuracy was estimated using the marginal and conditional R^2 values (R^2m and R^2c, respectively) as defined by Nakagawa and Schielzeth (2013). For each factor, *F*, we obtained its marginal persistence estimate, $Peff_F^m$ (i.e., where the persistence estimates of the remaining covariates are set to their mean values) and the range of persistence estimates, $Peff_F^{\Delta}$, among the varying levels of *F*. The effect size of the respective factor *F* ($Peff_F$) was therefore obtained using the $Peff_F^{\Delta}/Peff_F^m$ ratio with confidence intervals assessed through the Fieller's estimation method for the ratio of two Gaussian distributions (Beyene and Moineddin 2005).

a) All conflict levels

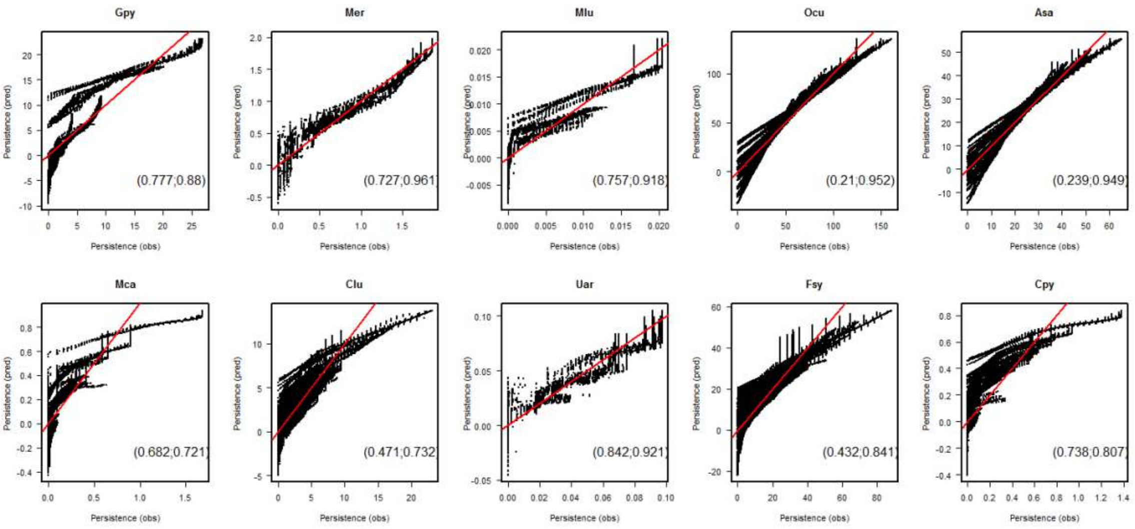

b) 1st quartile (Q1)

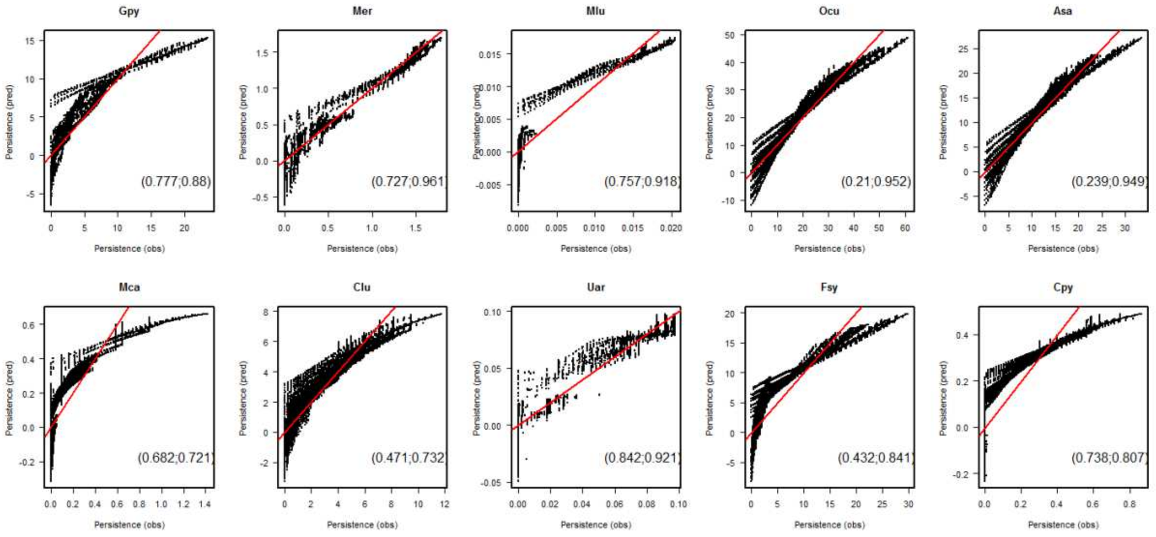

(it continues)

c) 2nd quartile (Q2)

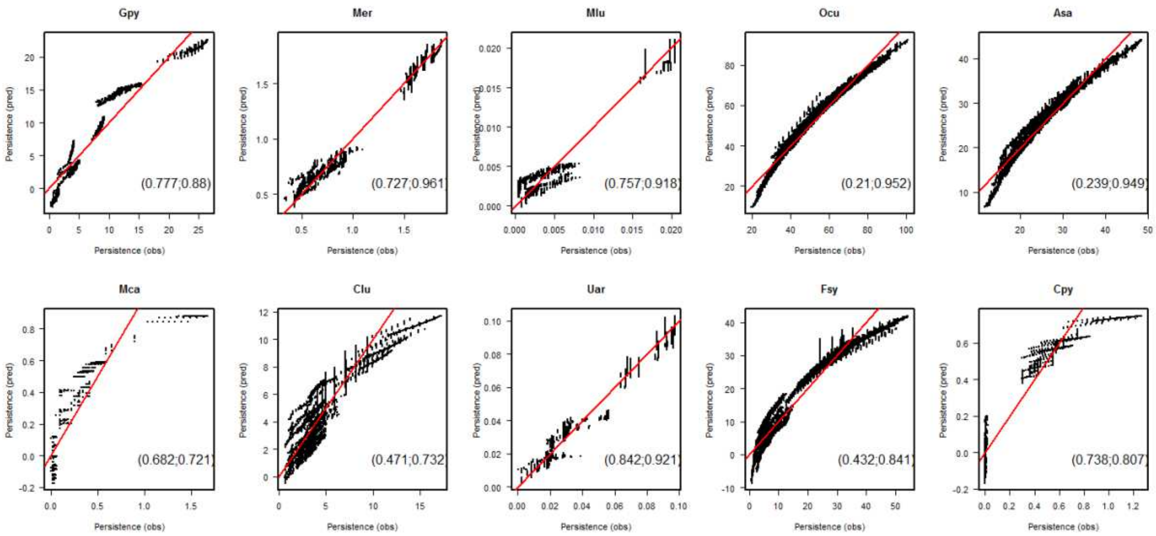

d) 3rd quartile (Q3)

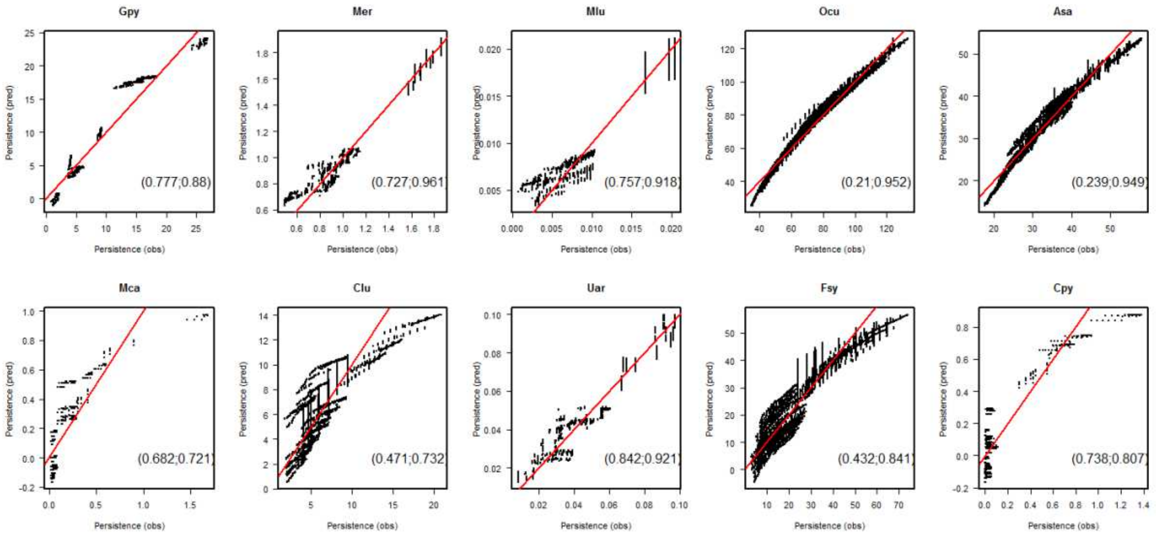

(it continues)

e) 4th quartile (Q4)

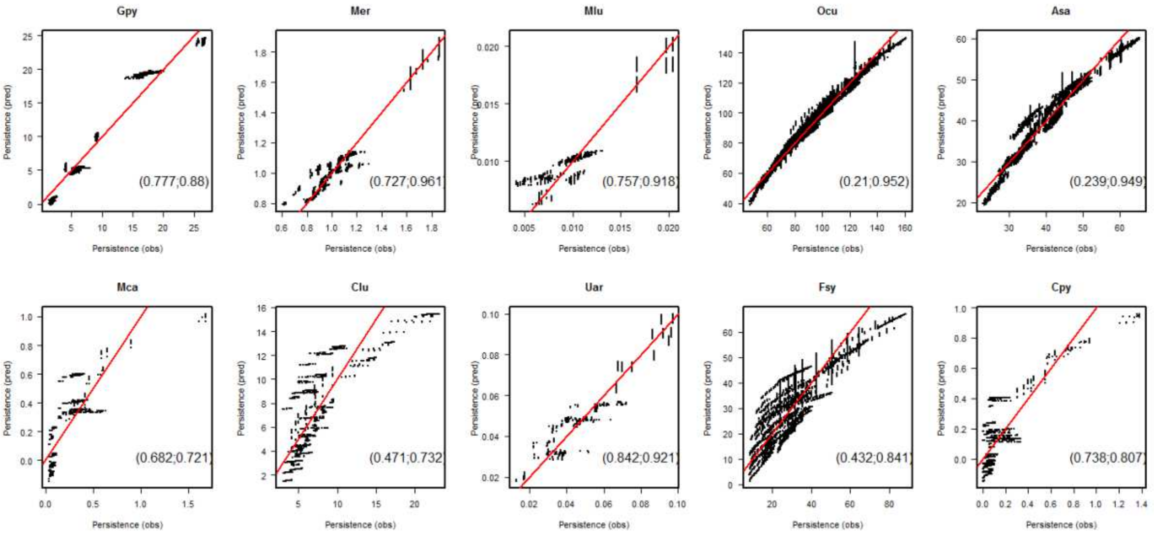

Figure S8 – Observed and predicted species persistence scores within CCCs, ranging different levels of conflict with socio-economic activities. **a)** all conflict levels; **b),c),d)** and **e)** 1st, 2nd, 3rd and 4th quartiles, respectively. Pairs of values refer to the marginal and conditional R squares. ***Gpy****: Galemys pyrenaicus;* ***Mer****: Mustela erminea;* ***Mlu****: Mustela lutreola;* ***Ocu****; Oryctolagus cunniculus;* ***Asa****: Arvicola sapidus;* ***Mca****: Microtus cabrerae;* ***Clu****; Canis Lupus;* ***Uar****. Ursus arctus;* ***Fsy****: Felix sylvestris;* ***Cpy****: Capra pyrenaica.*

a) All conflict levels

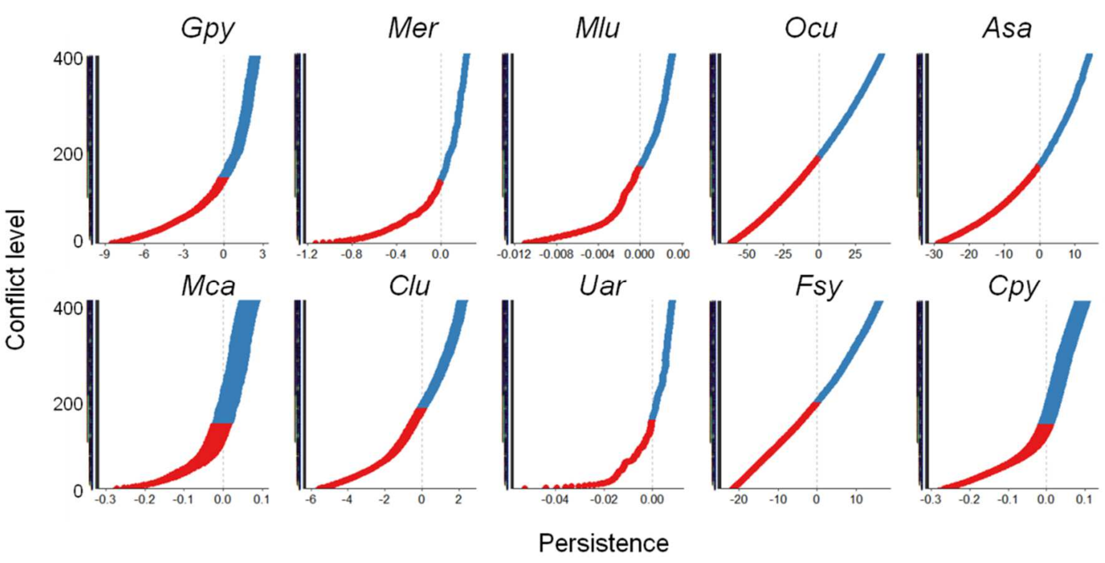

b) 1st quartile (Q1)

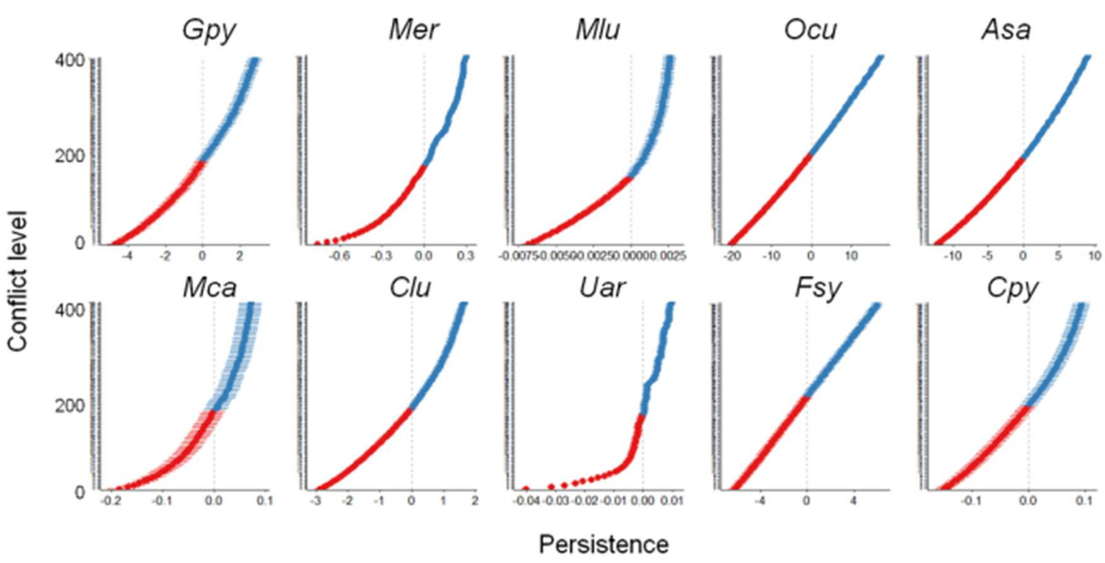

(it continues)

c) 2nd quartile (Q2)

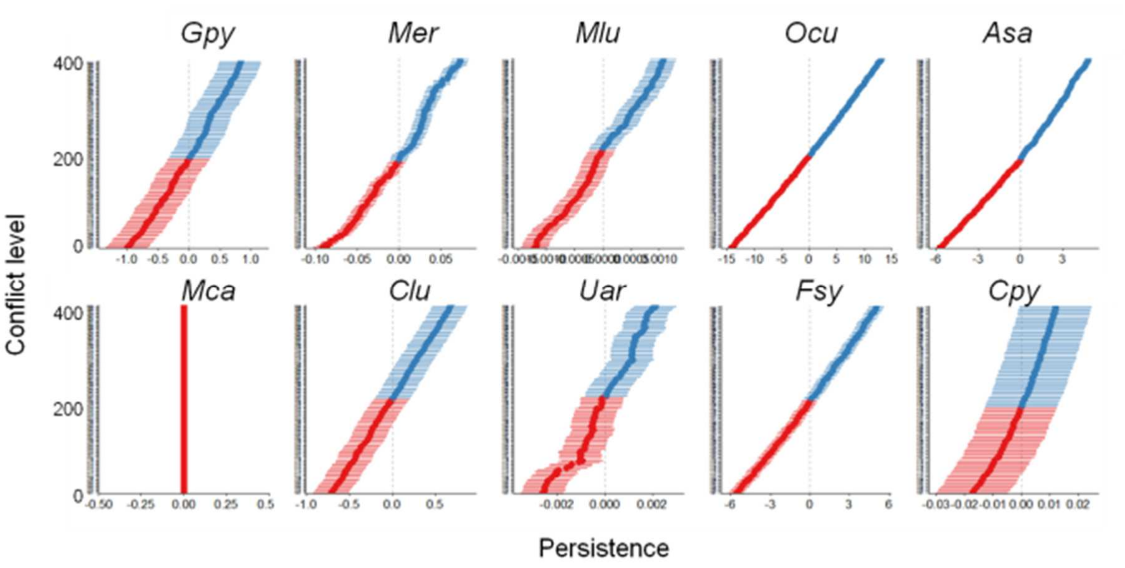

d) 3rd quartile (Q3)

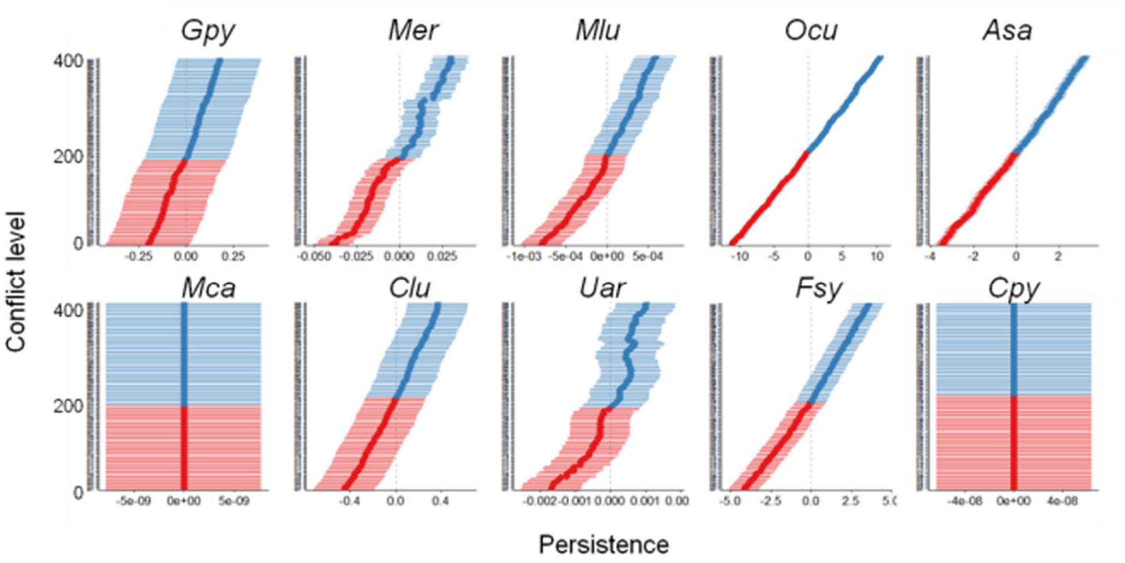

(it continues)

e) 4th quartile (Q4)

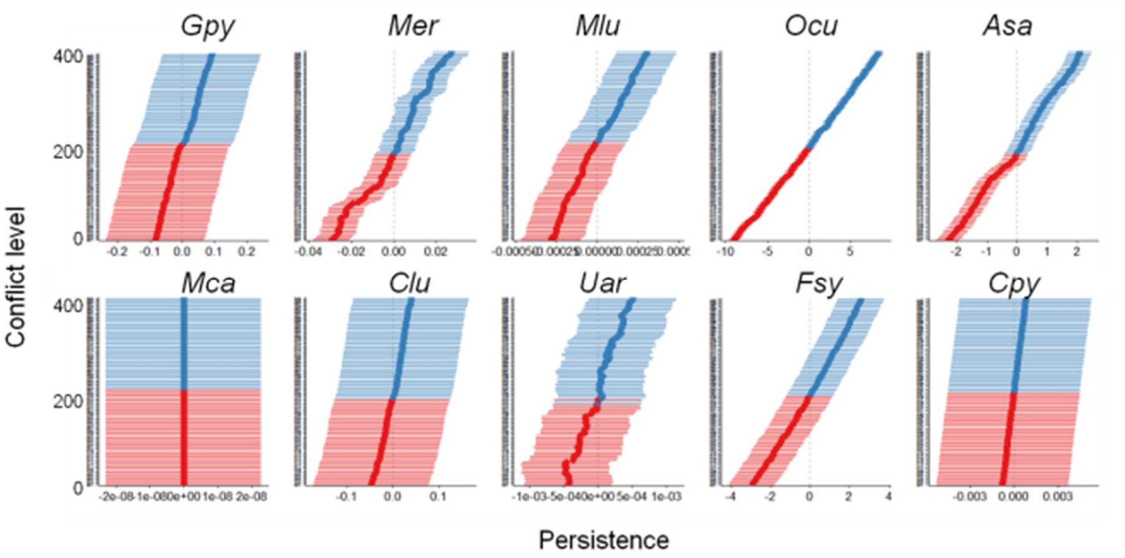

Figure S9 – The predicted species persistence expectancies in CCCs, only respecting the variation of levels of conflict with socio-economic activities (random effect in a linear mixed model). **a)** all conflict levels; **b),c),d)** and **e)** 1st, 2nd, 3rd and 4th quartiles, respectively. Red lines refer to negative persistence values predictions. ***Gpy***: *Galemys pyrenaicus;* ***Mer***: *Mustela erminea;* ***Mlu***: *Mustela lutreola;* ***Ocu***; *Oryctolagus cunniculus;* ***Asa***: *Arvicola sapidus;* ***Mca***: *Microtus cabrerae;* ***Clu***; *Canis lupus;* ***Uar***. *Ursus arctus;* ***Fsy***: *Felix sylvestris;* ***Cpy***: *Capra pyrenaica.*

a) 1st quartile (Q1)

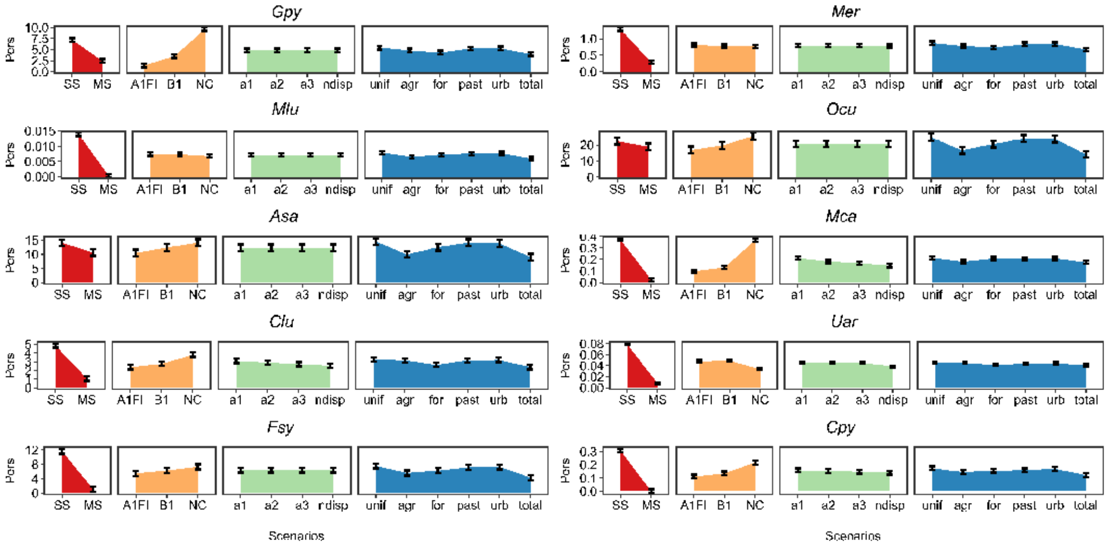

b) 2nd quartile (Q2)

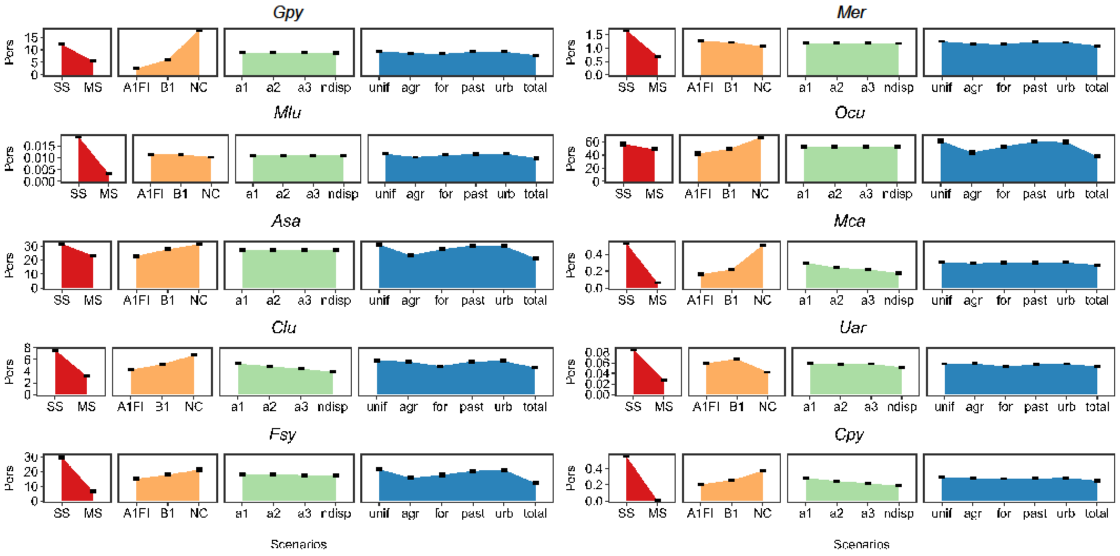

(it continues)

c) 3rd quartile (Q3)

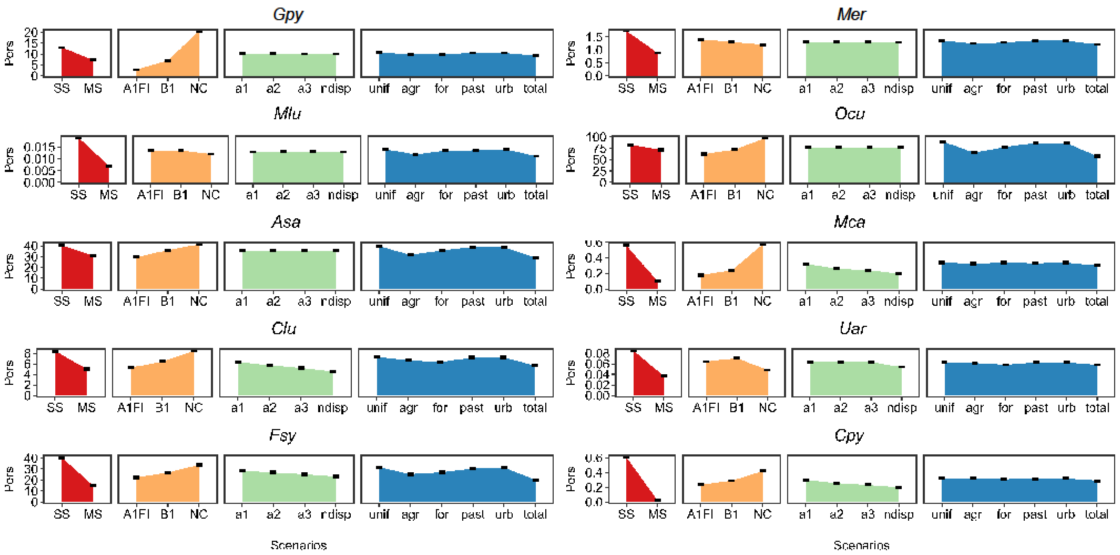

d) 4th quartile (Q4)

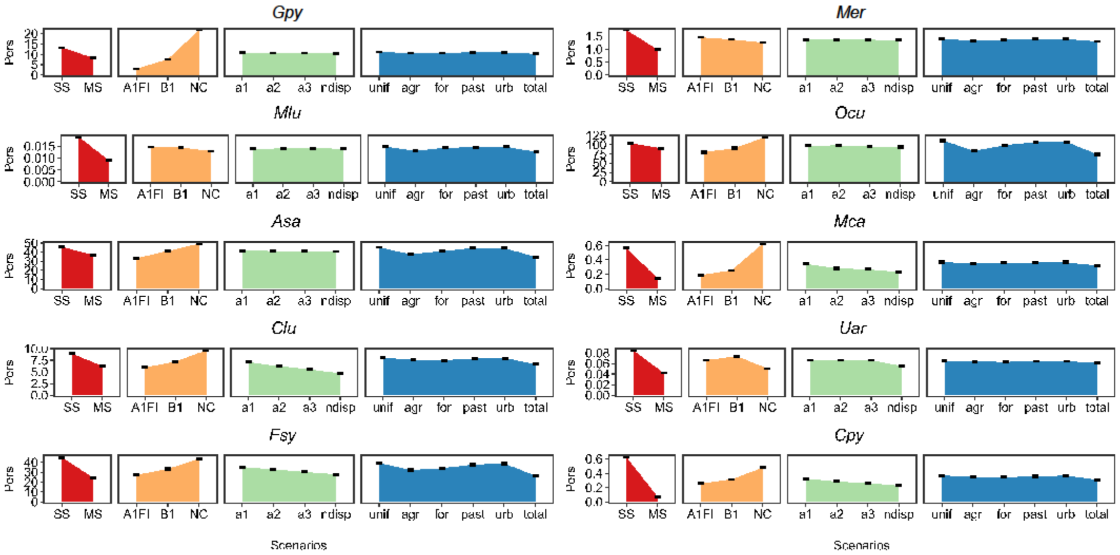

Figure S10 – Variation of species persistence scores with changes in the tested factors. Red polygons respect to variations within planning design (SS: single species; MS: multiple species). Yellow polygons respect to climate scenario (A1FI, B1 and NC: no-change). Green polygons ms respect to species dispersal ability (ndisp: non-dispersal; a1, a2, a3: α1, α2 and α3). Blue polygons respect conflict types (unif: single-uniform; agr: agriculture; for: forestry; past; pasture lands; urb: urban areas and total conflict). Analyses were undertaken for distinct levels of conflict: **a), b), c)** and **d)** 1st, 2nd, 3rd and 4th quartiles, respectively. ***Gpy***: *Galemys pyrenaicus;* ***Mer***: *Mustela erminea;* ***Mlu***: *Mustela lutreola;* ***Ocu***; *Oryctolagus cunniculus;* ***Asa***: *Arvicola sapidus;* ***Mca***: *Microtus cabrerae;* ***Clu***; *Canis lupus;* ***Uar***. *Ursus arctus;* ***Fsy***: *Felix sylvestris;* ***Cpy***: *Capra pyrenaica.*

2) References in Appendix 2

Beyene J., Moineddin R. (2005) Methods for confidence interval estimation of a ratio parameter with application to location quotients. *BMC Medical Research Methodology* **5**, 1.

Nakagawa S., Schielzeth H. (2013) A general and simple method for obtaining R2 from generalized linear mixed-effects models. *Methods in Ecology and Evolution* **4**, 133-142.

ndisp.unif

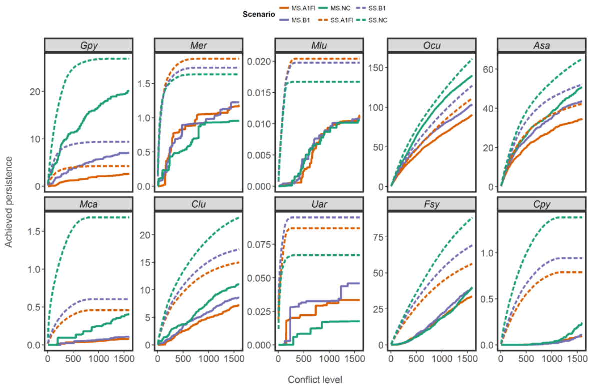

ndisp.agr

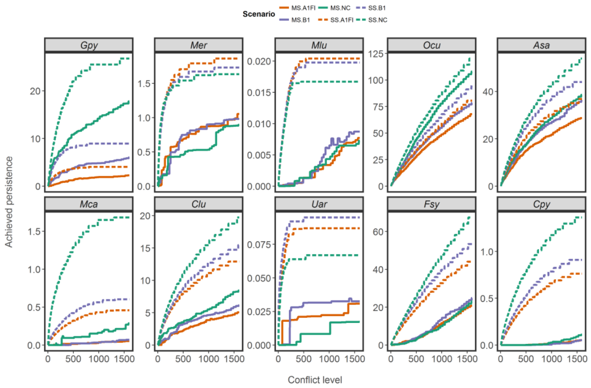

ndisp.for

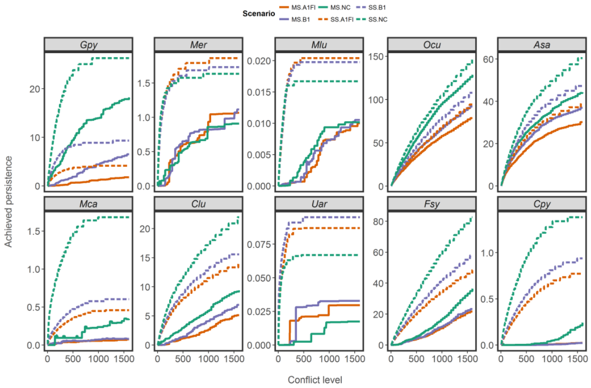

ndisp.past

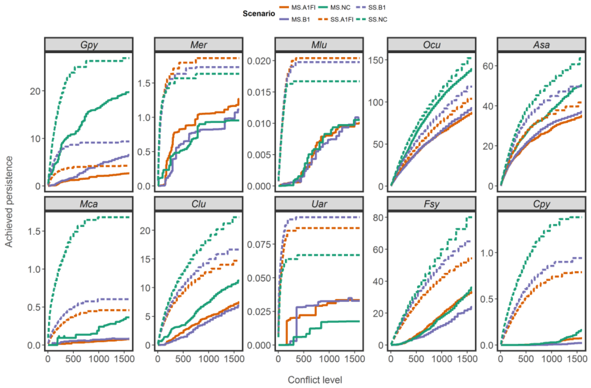

ndisp.urb

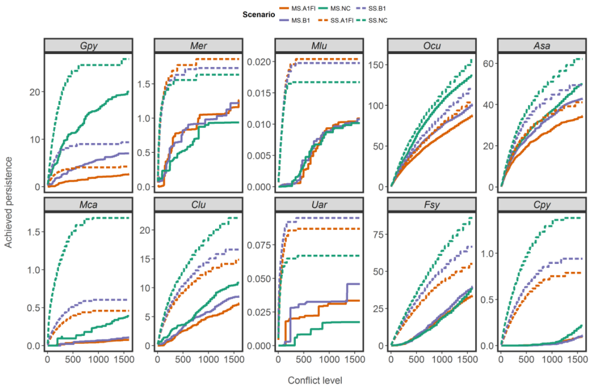

ndisp.total

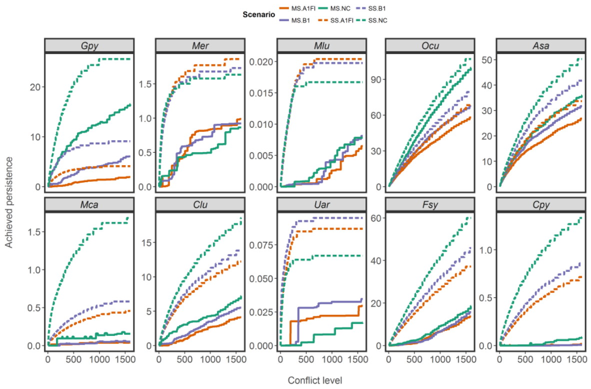

α1. unif

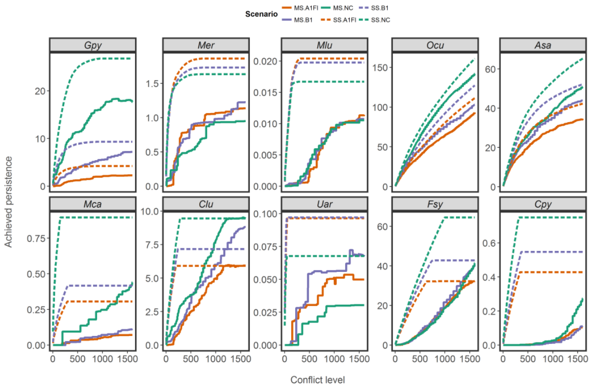

α1. agr

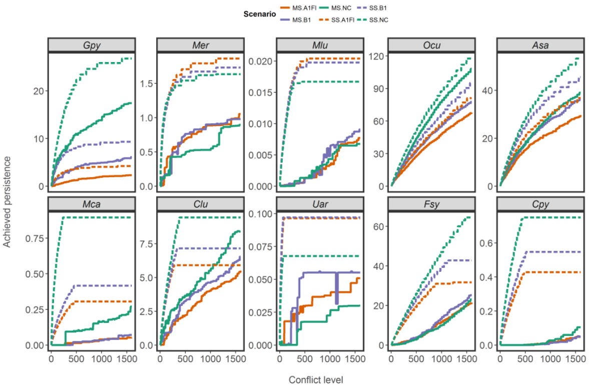

α1. for

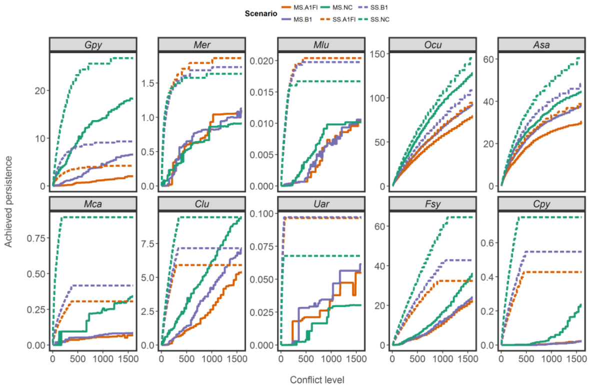

α1. past

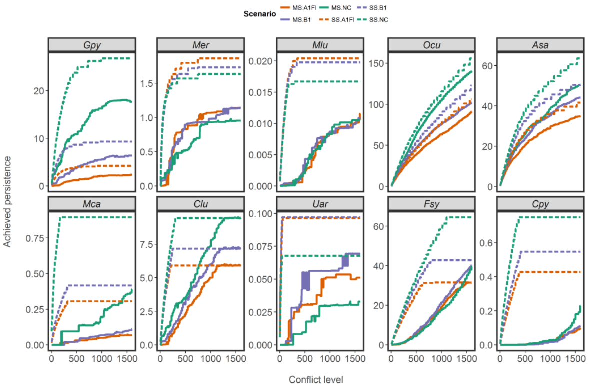

α1. urb

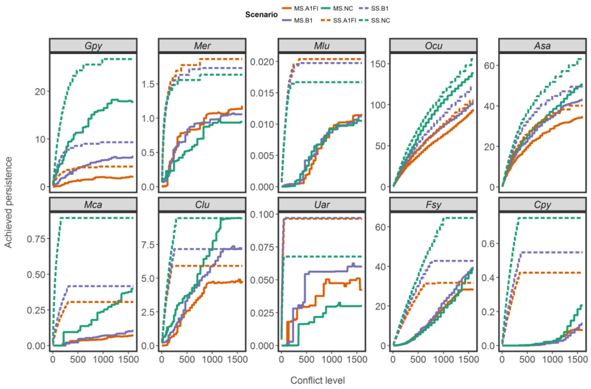

α1. total

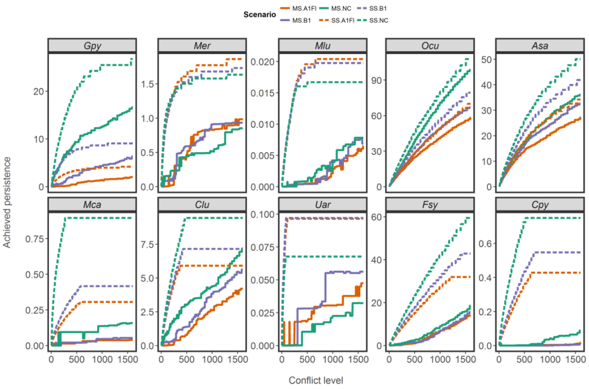

α2. unif

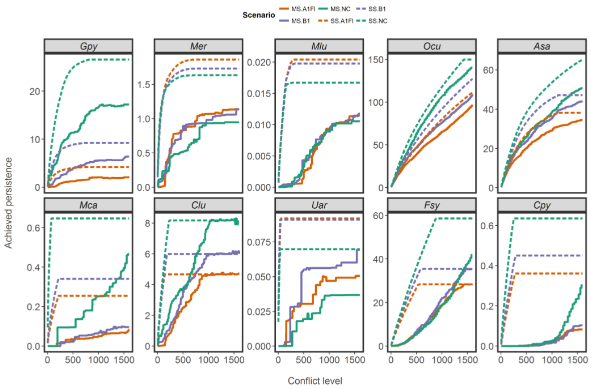

α2. agr

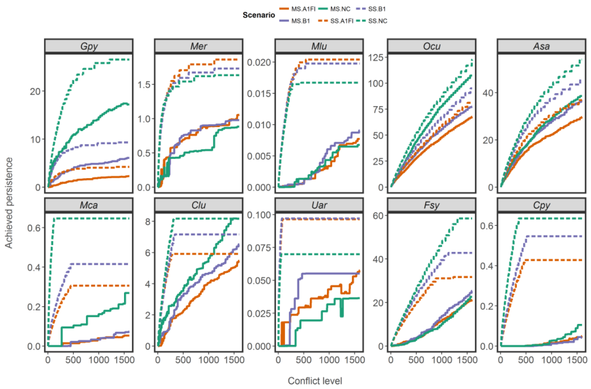

α2. for

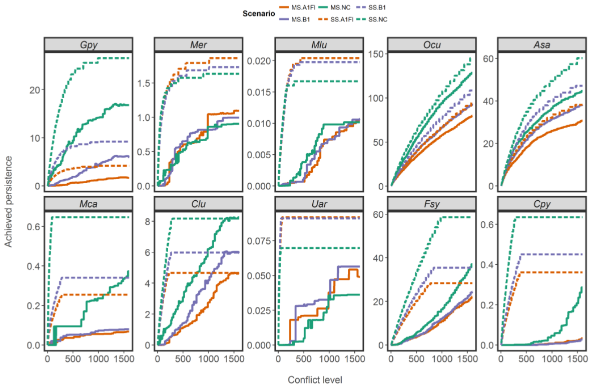

α2. past

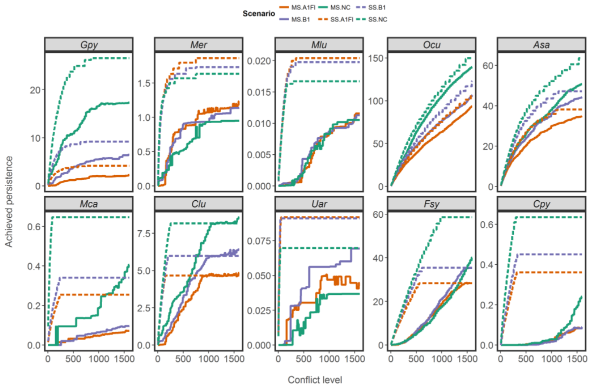

α2. urb

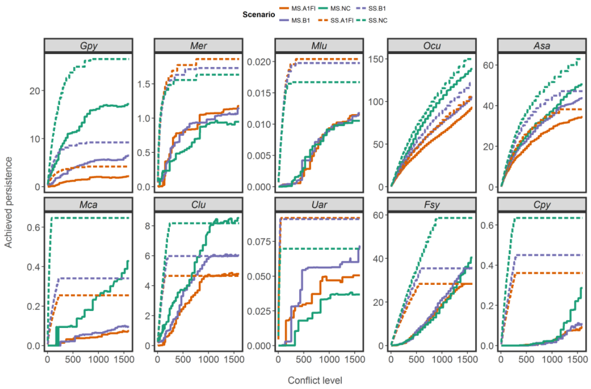

α2. total

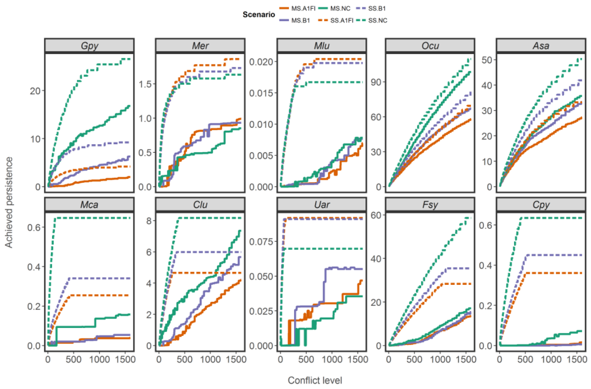

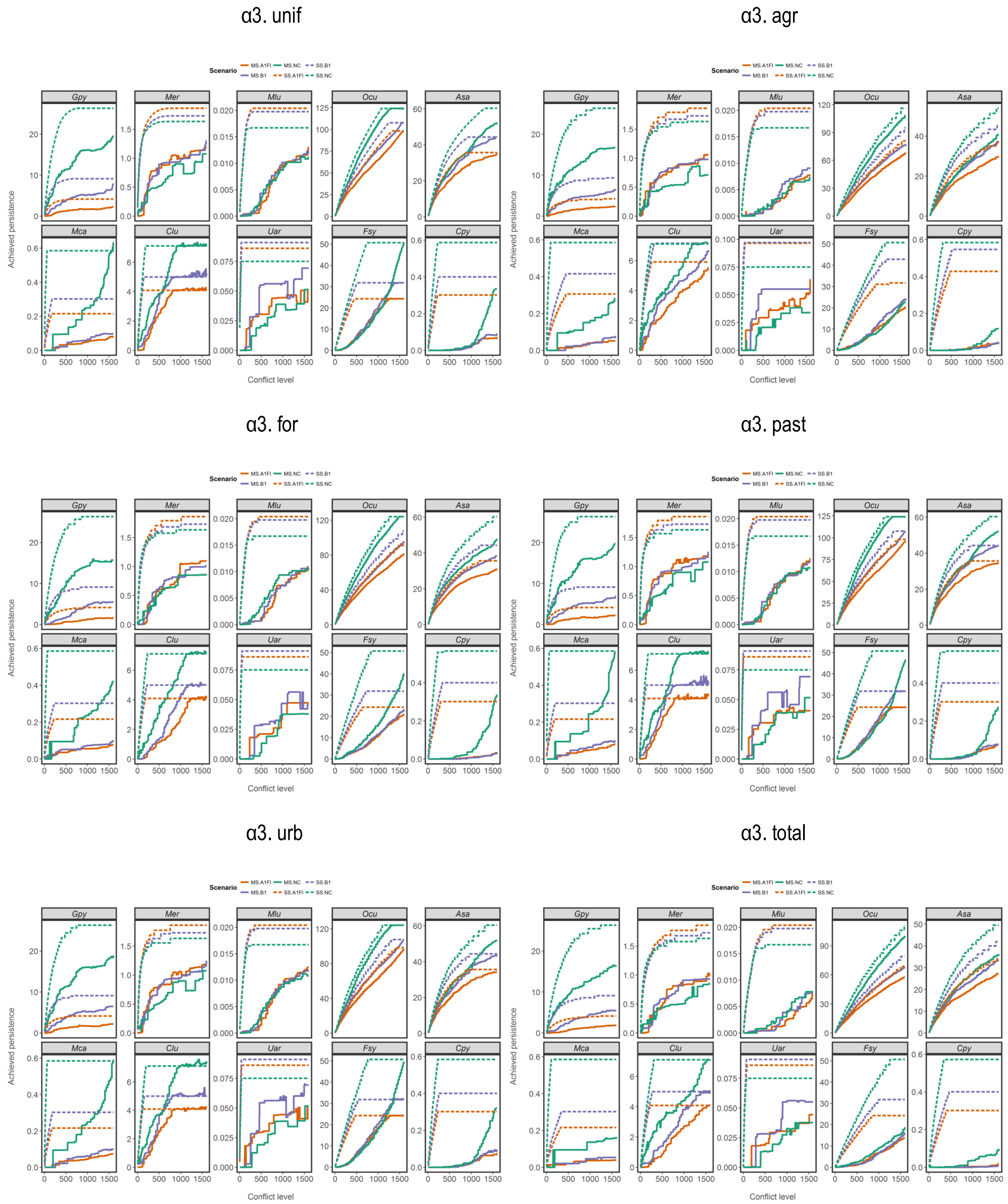

Figure S11 – Variation of species persistence scores with varying conflict levels. Analyses were undertaken for several dispersal ability assumptions (ndisp, α1, α2 and α3) and conflict types (unif: unitary-uniform, agr: agriculture; for: forestry; past; pasture land; urb; urban areas and total): Colored lines refer to runs with varying climate scenarios (A1FI, B1 and NC: no change) and planning designs (SS: single species; MS: multiple species). ***Gpy***: *Galemys pyrenaicus;* ***Mer***: *Mustela erminea;* ***Mlu***: *Mustela lutreola;* ***Ocu****; Oryctolagus cunniculus;* ***Asa***: *Arvicola sapidus;* ***Mca***: *Microtus cabrerae;* ***Clu***; *Canis lupus;* ***Uar***. *Ursus arctus;* ***Fsy***: *Felix sylvestris;* ***Cpy***: *Capra pyrenaica.*

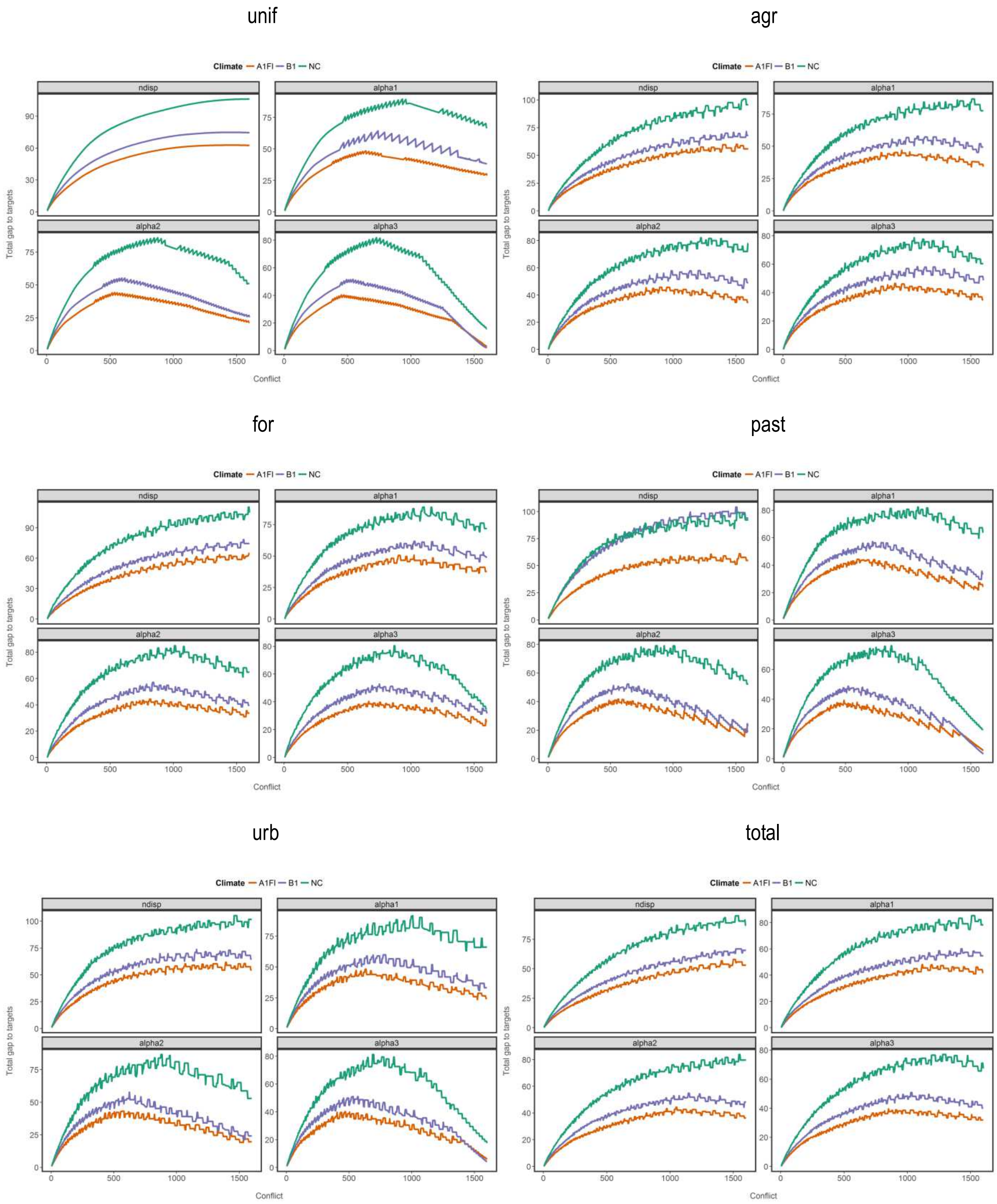

Figure S12a –The summed gaps to the targeted persistence scores (from SS planning designs) for the ten analysed species, running MS designs with varying conflict levels. Analyses were made for different conflict types (unif: unitary-uniform, agr: agriculture; for: forestry; past; pasture land, urb: urban areas and total). Colored lines represent distinct scenarios of climate (A1FI, B1 and NC: no-change).

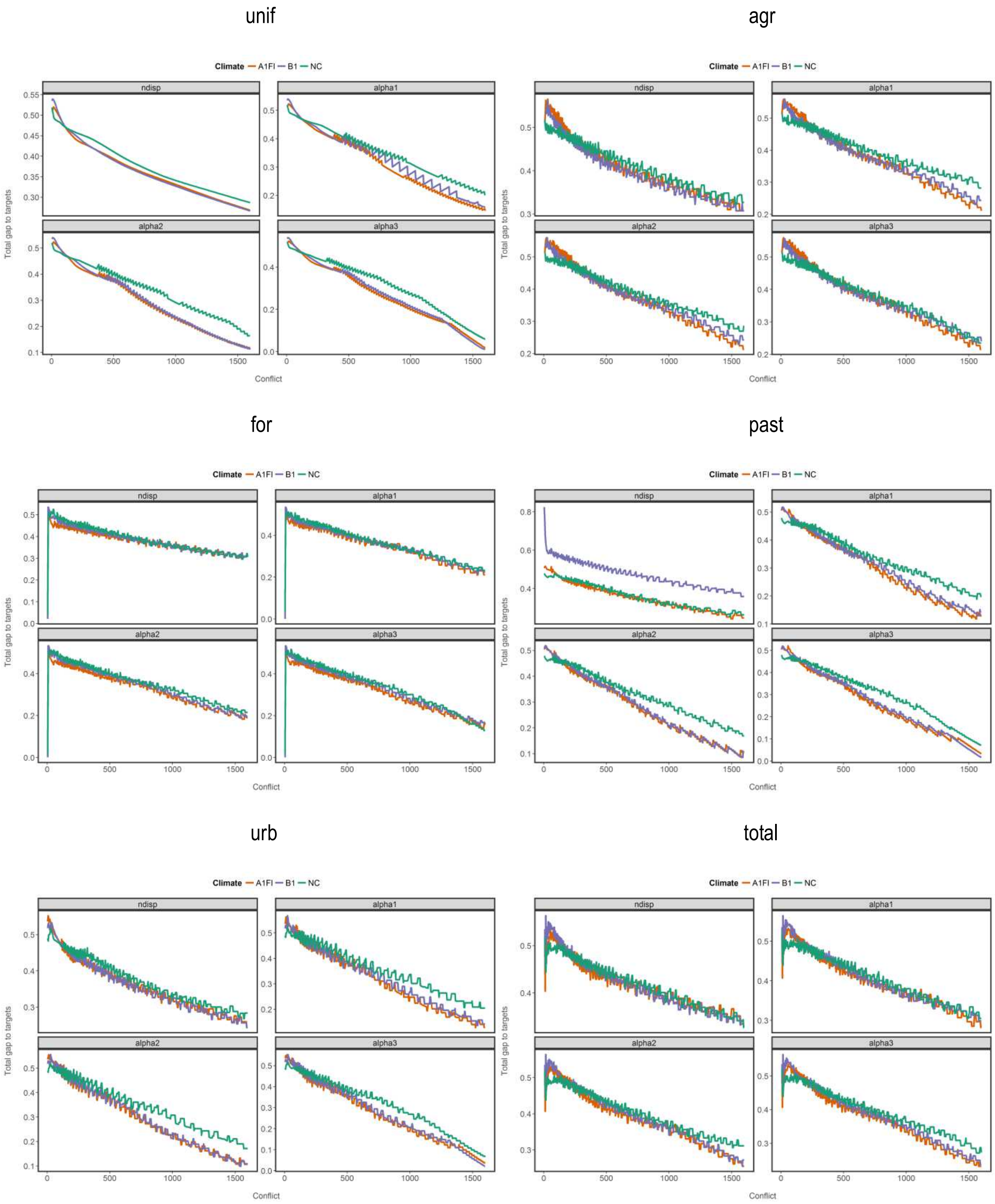

Figure S12b – The summed gaps to the targeted persistence scores (relative values to) of the ten analysed species, when running MS designs with varying conflict levels. Analyses were made for different conflict types (unif: unitary-uniform, agr: agriculture; for: forestry; past; pasture land, urb: urban areas and total). Colored lines represent distinct scenarios of climate (A1FI, B1 and NC: no-change).

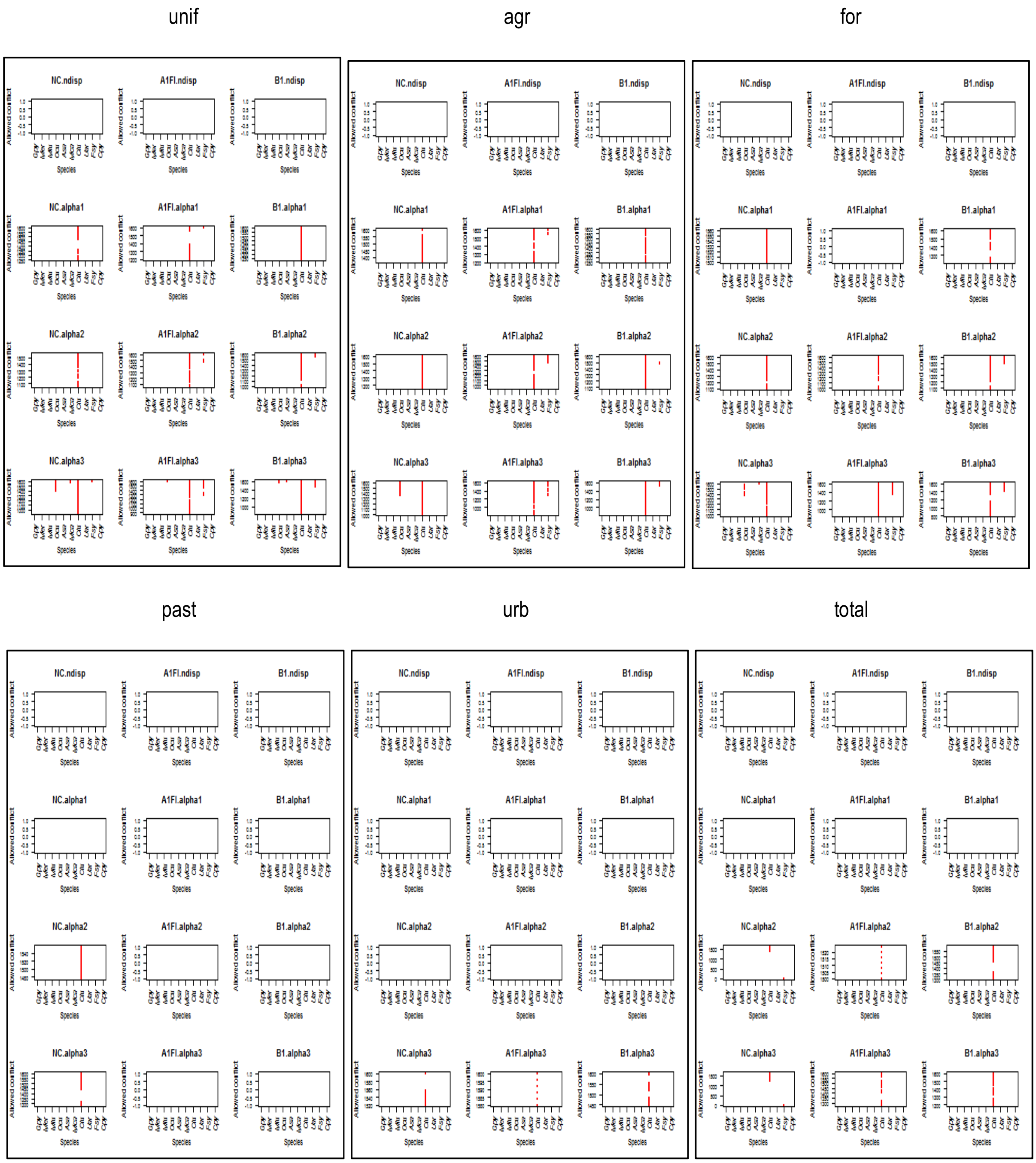

Figure S13 – The MS planning design solutions (red dots) in which each species is adequately covered (i.e. the required persistence target was reached) in the CCCs identified for a range of allowed conflict levels. Solutions were obtained for distinct conflict types (unif: unitary-uniform; agr: agriculture; for: forestry; past: pasture land; urb: urban area and total), for distinct climate scenarios (A1FI, B1 and NC: no change) and dispersal ability assumptions (ndisp: non-dispersal, alpha1, alpha2 and alpha3). ***Gpy***: *Galemys pyrenaicus;* ***Mer***: *Mustela erminea;* ***Mlu***: *Mustela lutreola;* ***Ocu***; *Oryctolagus cunniculus;* ***Asa***: *Arvicola sapidus;* ***Mca***: *Microtus cabrerae;* ***Clu***; *Canis lupus;* ***Uar***. *Ursus arctus;* ***Fsy***: *Felix sylvestris;* ***Cpy***: *Capra pyrenaica.*

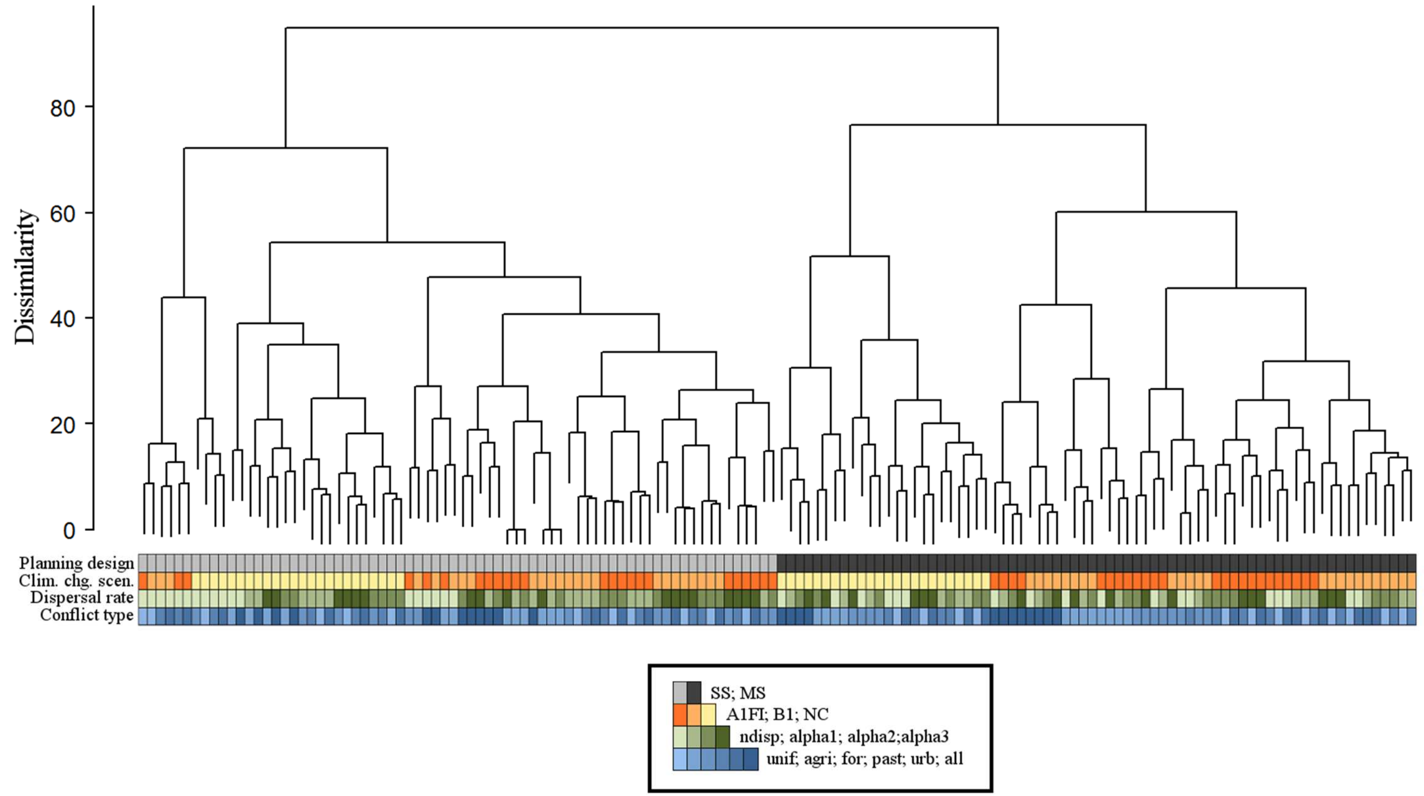

Figure S14 – Cluster analysis (UPGMA) for the dissimilarities between the areas defining CCCs, in which the mean number of species in each planning unit for each time period (summed across all the tested conflict levels) is recorded. Solutions were obtained varying planning design (SS: single species; MS: multiple species), climate scenario (A1FI, B1, NC: no change), dispersal ability assumptions (ndisp: non-dispersal, alpha1, alpha2 and alpha3) and conflict types (unif: unitary-uniform; agr: agriculture; for: forestry; past: pasture land; urb: urban areas and total)
