# Supplementary material for: A quantitative analysis on the effects of critical factors limiting the effectiveness of species conservation in future time
Source: Ecol Evol. 2018 Feb 24;8(6):3457–67. doi: 10.1002/ece3.3788 (PMC5869367; doi:10.1002/ece3.3788)
Supplement: Supplementary file 3 [file ECE3-8-3457-s003.pdf]

SUPPLEMENTARY MATERIAL

APPENDIX 3

- TABLES -

CONTENTS

Table S3a ----- 2

Table S3b ----- 3-4

Table S4a ----- 5

Table S4b ----- 6-7

Table S5 ----- 8

Table S3a – Summary of mixed linear model results for additive effects of planning design, climate scenario, dispersal ability and socio-economic activities conflict types over species persistence in CCCs. SS for sum of squares, MS for mean square, F for the F-test value, R2m and R2c for the goodness-of-fit for the marginal and marginal with conditional model components. For each species, underlined text marks the factor presenting the highest MS value.

Species	Factor	df	SS	MS	F	R2m	R2c
Gpy	**Plan**	1	436867.18	436867.18	58658.88	0.78	0.88
	**Climate**	2	2337140.96	**1168570.48**	156905.91		
	**Dispersal**	3	39.00	13.00	1.75		
	**Confl**	5	12066.50	2413.30	324.04		
Mer	**Plan**	1	11476.29	**11476.29**	1026091.96	0.73	0.96
	**Climate**	2	254.43	127.21	11374.11		
	**Dispersal**	3	0.26	0.09	7.62		
	**Confl**	5	177.37	35.47	3171.74		
Mlu	**Plan**	1	2.34	2.34	515717.92	0.76	0.92
	**Climate**	2	0.02	0.01	2200.12		
	**Dispersal**	3	0.00	0.00	3.69		
	**Conf**	5	0.04	0.01	1840.03		
Ocu	**Plan**	1	1140448.64	1140448.64	20443.07	0.21	0.95
	**Climate**	2	7634354.46	**3817177.23**	68424.68		
	**Dispersal**	3	3397.83	1132.61	20.30		
	**Confl**	5	5400599.72	1080119.94	19361.65		
Asa	**Plan**	1	875077.04	**875077.04**	93726.02	0.24	0.95
	**Climate**	2	958623.86	479311.93	51337.20		
	**Dispersal**	3	413.58	137.86	14.77		
	**Conf**	5	707447.92	141489.58	15154.39		
Mca	**Plan**	1	2576.62	**2576.62**	82847.48	0.68	0.72
	**Climate**	2	1461.97	730.98	23503.71		
	**Dispersal**	3	333.70	111.23	3576.55		
	**Confl**	5	11.62	2.32	74.73		
Clu	**Plan**	1	184894.40	**184894.40**	51178.04	0.47	0.73
	**Climate**	2	71941.59	35970.79	9956.57		
	**Dispersal**	3	96521.29	32173.76	8905.57		
	**Confl**	5	12742.50	2548.50	705.41		
Uar	**Plan**	1	43.07	**43.07**	544324.10	0.84	0.92
	**Climate**	2	4.76	2.38	30055.45		
	**Dispersal**	3	0.75	0.25	3174.67		
	**Confl**	5	0.19	0.04	483.57		
Fsy	**Plan**	1	5857637.71	**5857637.71**	124232.37	0.43	0.84
	**Climate**	2	783315.45	391657.73	8306.52		
	**Dispersal**	3	125401.39	41800.46	886.53		
	**Confl**	5	612957.76	122591.55	2600.00		
Cpy	**Plan**	1	3578.52	**3578.52**	191861.10	0.74	0.81
	**Climate**	2	294.62	147.31	7897.90		
	**Dispersal**	3	230.81	76.94	4124.84		
	**Confl**	5	13.24	2.65	142.01		

***Gpy**: Galemys pyrenaicus; **Mer**: Mustela erminea; **Mlu**: Mustela lutreola; **Ocu**; Oryctolagus cunniculus; **Asa**: Arvicola sapidus; **Mca**: Microtus cabrerae; **Clu**; Canis lupus; **Uar**. Ursus arctus; **Fsy**: Felix sylvestris; **Cpy**: Capra pyrenaica.*

Table S3b – Summary of mixed linear model results for additive effects of planning design, climate scenario, dispersal ability and socio-economic activities conflict types over species persistence in CCCs. SS for sum of squares, MS for mean square, F for the F-test value, R2m and R2c for the goodness-of-fit for the marginal and marginal with conditional model components. Values refer to models applied over the 1st (Q1) 2nd (Q2), 3rd (Q3) and 4th (Q4) conflict level quartiles.

			Q1					Q2				
**Species**	**Factor**	**df**	**SS**	**MS**	**F**	**R2m**	**R2c**	**SS**	**MS**	**F**	**R2m**	**R2c**
Gpy	**Plan**	**1**	80348.92	80348.92	14593.50	0.64	0.80	157636.25	157636.25	32998.90	0.91	0.92
	**Climate**	2	172037.30	**86018.65**	15623.27			615166.03	**307583.02**	64388.11		
	**Dispersal**	3	2.93	0.98	0.18			11.94	3.98	0.83		
	**Confl**	5	4210.05	842.01	152.93			4309.40	861.88	180.42		
Mer	**Plan**	1	3644.99	**3644.99**	198039.13	0.76	0.95	3398.76	**3398.76**	871677.28	0.98	0.98
	**Climate**	2	5.11	2.56	138.84			105.97	52.98	13588.48		
	**Dispersal**	3	0.10	0.03	1.80			0.08	0.03	6.64		
	**Confl**	5	70.93	14.19	770.77			48.77	9.75	2501.58		
Mlu	**Plan**	1	0.66	**0.66**	95193.77	0.77	0.89	0.89	**0.89**	413632.50	0.96	0.97
	**Climate**	2	0.00	0.00	85.87			0.00	0.00	1097.91		
	**Dispersal**	3	0.00	0.00	0.00			0.00	0.00	0.19		
	**Conf**	5	0.01	0.00	194.85			0.01	0.00	782.56		
Ocu	**Plan**	1	46206.67	46206.67	4242.87	0.20	0.93	213847.69	213847.69	26165.25	0.74	0.97
	**Climate**	2	186744.83	**93372.41**	8573.80			1513951.02	**756975.51**	92619.44		
	**Dispersal**	3	4.93	1.64	0.15			5.63	1.88	0.23		
	**Confl**	5	229776.21	45955.24	4219.78			1157063.85	231412.77	28314.42		
Asa	**Plan**	1	45603.94	**45603.94**	14190.35	0.20	0.94	263785.09	**263785.09**	106937.01	0.79	0.96
	**Climate**	2	31990.71	15995.36	4977.20			173061.67	86530.83	35079.12		
	**Dispersal**	3	0.66	0.22	0.07			111.16	37.05	15.02		
	**Conf**	5	67635.54	13527.11	4209.16			204385.69	40877.14	16571.37		
Mca	**Plan**	1	441.99	**441.99**	23682.85	0.67	0.73	789.16	**789.16**	26513.42	0.74	0.74
	**Climate**	2	206.29	103.14	5526.60			333.43	166.71	5601.09		
	**Dispersal**	3	8.56	2.85	152.93			90.69	30.23	1015.64		
	**Confl**	5	3.22	0.64	34.49			2.17	0.43	14.60		
Clu	**Plan**	1	51795.58	**51795.58**	59813.43	0.62	0.87	71295.54	**71295.54**	42028.98	0.79	0.81
	**Climate**	2	5283.16	2641.58	3050.49			15630.98	7815.49	4607.26		
	**Dispersal**	3	402.56	134.19	154.96			14001.90	4667.30	2751.39		
	**Confl**	5	1547.37	309.47	357.38			3262.40	652.48	384.64		
Uar	**Plan**	1	17.97	**17.97**	211640.72	0.89	0.94	11.73	**11.73**	350922.61	0.96	0.97
	**Climate**	2	0.70	0.35	4122.29			1.54	0.77	23069.31		
	**Dispersal**	3	0.11	0.04	424.86			0.12	0.04	1231.21		
	**Confl**	5	0.05	0.01	109.99			0.10	0.02	623.06		
Fsy	**Plan**	1	404285.52	**404285.52**	40818.39	0.57	0.81	2012539.88	**2012539.88**	157597.64	0.88	0.93
	**Climate**	2	8281.48	4140.74	418.07			91548.41	45774.20	3584.48		
	**Dispersal**	3	0.99	0.33	0.03			1611.63	537.21	42.07		
	**Confl**	5	18509.26	3701.85	373.75			163688.19	32737.64	2563.61		
Cpy	**Plan**	1	340.82	**340.82**	38612.44	0.65	0.78	1082.25	**1082.25**	111420.23	0.89	0.90
	**Climate**	2	30.18	15.09	1709.70			67.76	33.88	3488.20		
	**Dispersal**	3	0.31	0.10	11.54			38.14	12.71	1308.96		
	**Confl**	5	4.41	0.88	99.93			2.42	0.48	49.85		

***Gpy**: Galemys pyrenaicus; **Mer**: Mustela erminea; **Mlu**: Mustela lutreola; **Ocu**; Oryctolagus cunniculus; **Asa**: Arvicola sapidus; **Mca**: Microtus cabrerae; **Clu**; Canis lupus; **Uar**. Ursus arctus; **Fsy**: Felix sylvestris; **Cpy**: Capra pyrenaica.*

(it continues in the next page)

			Q3					Q4				
Species	Factor	df	SS	MS	F	R2m	R2c	SS	MS	F	R2m	R2c
Gpy	**Plan**	1	119225.25	119225.25	32509.21	0.95	0.95	87696.12	87696.12	31996.07	0.96	0.96
	**Climate**	2	819047.95	**409523.98**	111665.11			909190.86	**454595.43**	165859.89		
	**Dispersal**	3	26.34	8.78	2.39			165.84	55.28	20.17		
	**Conf**	5	3227.46	645.49	176.01			1130.45	226.09	82.49		
Mer	**Plan**	1	2550.35	**2550.35**	583726.25	0.98	0.98	2032.32	**2032.32**	700724.22	0.98	0.98
	**Climate**	2	101.31	50.65	11593.93			93.33	46.66	16088.85		
	**Dispersal**	3	0.17	0.06	13.16			0.02	0.01	2.65		
	**Conf**	5	52.79	10.56	2416.72			20.19	4.04	1391.95		
Mlu	**Plan**	1	0.51	**0.51**	199279.20	0.93	0.94	0.35	**0.35**	215172.70	0.94	0.94
	**Climate**	2	0.01	0.00	1354.01			0.01	0.00	2864.48		
	**Dispersal**	3	0.00	0.00	2.74			0.00	0.00	18.96		
	**Conf**	5	0.02	0.00	1399.61			0.01	0.00	1275.53		
Ocu	**Plan**	1	440059.75	440059.75	49055.15	0.89	0.98	632139.07	632139.07	40964.22	0.93	0.97
	**Climate**	2	3226999.09	**1613499.55**	179862.97			4274719.25	**2137359.62**	138506.33		
	**Dispersal**	3	189.10	63.03	7.03			16114.23	5371.41	348.08		
	**Conf**	5	2072364.03	414472.81	46202.87			2738047.96	547609.59	35486.49		
Asa	**Plan**	1	350953.72	**350953.72**	109619.63	0.90	0.96	303983.16	**303983.16**	52443.44	0.91	0.93
	**Climate**	2	339463.30	169731.65	53015.31			611447.46	**305723.73**	52743.72		
	**Dispersal**	3	340.01	113.34	35.40			814.21	271.40	46.82		
	**Conf**	5	242915.54	48583.11	15174.83			229219.37	45843.87	7909.03		
Mca	**Plan**	1	744.50	**744.50**	22204.15	0.73	0.73	631.00	**631.00**	19284.46	0.74	0.74
	**Climate**	2	434.03	217.02	6472.31			529.94	264.97	8097.95		
	**Dispersal**	3	143.60	47.87	1427.61			151.70	50.57	1545.41		
	**Conf**	5	2.37	0.47	14.12			4.67	0.93	28.52		
Clu	**Plan**	1	41512.77	**41512.77**	11707.56	0.69	0.69	26127.97	**26127.97**	5641.37	0.68	0.68
	**Climate**	2	24555.52	12277.76	3462.61			33256.14	16628.07	3590.22		
	**Dispersal**	3	42237.58	14079.19	3970.66			79263.43	**26421.14**	5704.67		
	**Conf**	5	5351.10	1070.22	301.83			3649.89	729.98	157.61		
Uar	**Plan**	1	8.27	**8.27**	224341.19	0.95	0.95	6.69	**6.69**	188965.97	0.94	0.94
	**Climate**	2	1.28	0.64	17363.15			1.38	0.69	19514.95		
	**Dispersal**	3	0.25	0.08	2247.71			0.31	0.10	2937.34		
	**Conf**	5	0.05	0.01	287.28			0.02	0.00	100.95		
Fsy	**Plan**	1	2371081.15	**2371081.15**	65174.31	0.83	0.85	1553027.48	**1553027.48**	26320.41	0.75	0.76
	**Climate**	2	323746.17	161873.09	4449.43			652848.18	326424.09	5532.17		
	**Dispersal**	3	48866.13	16288.71	447.73			209760.28	69920.09	1184.99		
	**Conf**	5	234018.83	46803.77	1286.50			294259.56	58851.91	997.41		
Cpy	**Plan**	1	1231.84	**1231.84**	75625.84	0.86	0.86	1101.25	**1101.25**	57191.02	0.83	0.83
	**Climate**	2	85.63	42.82	2628.58			129.19	64.59	3354.53		
	**Dispersal**	3	122.53	40.84	2507.56			165.97	55.32	2873.14		
	**Conf**	5	2.08	0.42	25.51			5.43	1.09	56.39		

Gpy: *Galemys pyrenaicus;* ***Mer***: *Mustela erminea;* ***Mlu***: *Mustela lutreola;* ***Ocu***; *Oryctolagus cunniculus;* ***Asa***: *Arvicola sapidus;* ***Mca***: *Microtus cabrerae;* ***Clu***; *Canis lupus;* ***Uar***. *Ursus arctus;* ***Fsy***: *Felix sylvestris;* ***Cpy***: *Capra pyrenaica.*

Table S4a – The effect sizes of planning designs (Plan), climate scenarios (Clim), dispersal abilities (Disp) and conflict level types (Confl) over species persistence expectancies within CCCs. See main text for the measuring of effect size (*Peff*) and confidence interval (CI). Underlined values mark the largest effect sizes for each species.

Sp	Factor	*Peff*	CI	t.test	df	*P*
Gpy	**Plan**	0.931	0.918-0.945	-9.69	57598	0.000
	**Clim**	<u>6.021</u>	5.854-6.198	245.35	38394	0.000
	**Disp**	0.020	0.010-0.029	-143.49	28796	0.000
	**Confl**	0.152	0.139-0.165	-92.66	19196	0.000
Mer	**Plan**	<u>1.282</u>	1.158-1.422	4.82	57474	0.000
	**Clim**	0.201	0.133-0.270	-19.44	32619	0.000
	**Disp**	0.020	-0.048-0.089	-25.04	21192	0.000
	**Confl**	0.179	0.119-0.239	-24.05	14198	0.000
Mlu	**Plan**	<u>2.588</u>	2.583-2.593	919.98	42393	0.000
	**Clim**	0.123	0.121-0.124	-1322.40	28198	0.000
	**Disp**	0.005	0.005-0.006	-1860.02	21171	0.000
	**Confl**	0.226	0.225-0.227	-1541.74	14051	0.000
Ocu	**Plan**	0.154	0.153-0.155	-1170.98	57598	0.000
	**Clim**	0.546	0.545-0.548	-445.24	38398	0.000
	**Disp**	0.010	0.009-0.011	-1035.58	28798	0.000
	**Confl**	<u>0.565</u>	0.563-0.568	-276.70	19196	0.000
Asa	**Plan**	0.308	0.306-0.311	-416.91	57597	0.000
	**Clim**	<u>0.415</u>	0.412-0.418	-273.38	38398	0.000
	**Disp**	0.009	0.006-0.011	-486.47	28798	0.000
	**Confl**	0.394	0.390-0.399	-196.96	19198	0.000
Mca	**Plan**	<u>5.109</u>	4.861-5.380	76.24	42286	0.000
	**Clim**	2.320	2.253-2.388	52.83	28479	0.000
	**Disp**	0.876	0.846-0.906	-7.89	21172	0.000
	**Confl**	0.185	0.165-0.206	-70.33	14214	0.000
Clu	**Plan**	<u>0.914</u>	0.894-0.934	-8.05	57598	0.000
	**Clim**	0.613	0.595-0.632	-33.65	38397	0.000
	**Disp**	0.758	0.735-0.782	-17.91	28798	0.000
	**Confl**	0.283	0.262-0.305	-48.15	19198	0.000
Uar	**Plan**	<u>1.908</u>	1.901-1.914	340.85	42395	0.000
	**Clim**	0.493	0.490-0.496	-351.33	28064	0.000
	**Disp**	0.173	0.171-0.175	-749.41	21070	0.000
	**Confl**	0.084	0.083-0.086	-1082.11	14112	0.000
Fsy	**Plan**	<u>1.724</u>	1.714-1.734	203.43	57595	0.000
	**Clim**	0.507	0.503-0.512	-169.83	38393	0.000
	**Disp**	0.203	0.199-0.207	-270.04	28790	0.000
	**Confl**	0.593	0.585-0.600	-88.03	19198	0.000
Cpy	**Plan**	<u>18.954</u>	15.536-24.267	71.70	42170	0.000
	**Clim**	0.804	0.754-0.855	-7.35	28136	0.000
	**Disp**	0.126	0.184-0.269	-12.04	21155	0.000
	**Confl**	0.201	0.171-0.231	-46.67	14098	0.000

Gpy*: Galemys pyrenaicus;* ***Mer****: Mustela erminea;* ***Mlu****: Mustela lutreola;* ***Ocu****; Oryctolagus cunniculus;* ***Asa****: Arvicola sapidus;* ***Mca****: Microtus cabrerae;* ***Clu****; Canis lupus;* ***Uar****. Ursus arctus;* ***Fsy****: Felix sylvestris;* ***Cpy****: Capra pyrenaica.*

Table S4b – The effect sizes of planning designs (Plan), climate scenarios (Clim), dispersal abilities (Disp) and conflict level types (Confl) over species persistence expectancies within CCCs. Values correspond to working scenarios using the 1st (Q1), 2nd (Q2), 3rd (Q3) and 4th (Q4) conflict level quartiles. See main text for the measuring of effect size (*Peff*) and confidence interval (CI). Underlined values mark the largest effect sizes for each species.

		Q1					Q2				
**Sp**	**Factor**	***Peff***	**CI**	**t.test**	**df**	***P***	***Peff***	**CI**	**t.test**	**df**	***P***
Gpy	**Plan**	1.894	1.798-1.999	26.30	14395	0.000	1.201	1.169-1.234	13.43	14398	0.000
	**Clim**	5.886	5.326-6.573	65.48	9598	0.000	6.244	5.894-6.637	124.42	9598	0.000
	**Disp**	-0.007	-0.041-0.026	-41.57	7198	0.000	0.011	0.000-0.038	-74.28	7192	0.000
	**Confl**	0.326	0.274-0.379	-18.68	4797	0.000	0.203	0.177-0.229	-43.67	4791	0.000
Mer	**Plan**	6.486	3.678-24.493	10.15	14376	0.000	1.395	1.383-1.407	72.22	10636	0.000
	**Clim**	0.005	-0.165-0.176	-9.67	8177	0.000	0.189	0.184-0.195	-272.58	6990	0.000
	**Disp**	0.004	-0.184-0.192	-9.35	5265	0.000	0.004	0.000-0.008	-422.10	5264	0.000
	**Confl**	0.496	0.290-0.711	-4.35	3538	0.000	0.159	0.155-0.163	-392.24	3564	0.000
Mlu	**Plan**	36.874	35.708-38.119	960.71	10557	0.000	4.970	4.969-4.972	13274.82	10688	0.000
	**Clim**	0.095	0.092-0.097	-548.99	7034	0.000	0.121	0.121-0.121	-11156.70	7034	0.000
	**Disp**	0.002	-0.001-0.004	-734.25	5228	0.000	0.002	0.002-0.002	-15408.04	5317	0.000
	**Confl**	0.319	0.317-0.322	-506.54	3482	0.000	0.202	0.202-0.203	-12710.60	3504	0.000
Ocu	**Plan**	0.184	0.178-0.190	-185.65	14397	0.000	0.157	0.154-0.159	-492.29	14398	0.000
	**Clim**	0.511	0.501-0.520	-81.37	9592	0.000	0.579	0.575-0.583	-172.88	9598	0.000
	**Disp**	0.002	0.000-0.016	-174.98	7197	0.000	-0.001	-0.004-0.002	-458.48	7197	0.000
	**Confl**	0.723	0.706-0.740	-28.19	4798	0.000	0.627	0.621-0.633	-101.27	4793	0.000
Asa	**Plan**	0.336	0.325-0.348	-83.42	14396	0.000	0.369	0.363-0.374	-174.10	14397	0.000
	**Clim**	0.346	0.332-0.360	-66.59	9598	0.000	0.373	0.367-0.380	-140.71	9598	0.000
	**Disp**	0.001	-0.012-0.014	-105.01	7196	0.000	0.000	-0.006-0.006	-230.40	7198	0.000
	**Confl**	0.633	0.607-0.659	-23.13	4796	0.000	0.471	0.460-0.481	-77.13	4798	0.000
Mca	**Plan**	11.598	9.117-15.841	34.75	10540	0.000	7.572	7.485-7.661	517.15	10663	0.000
	**Clim**	2.829	2.607-3.078	24.02	7184	0.000	2.123	2.109-2.138	193.34	7099	0.000
	**Disp**	0.232	0.166-0.299	-17.88	5255	0.000	0.230	0.220-0.239	-14.13	5395	0.000
	**Confl**	0.165	0.108-0.222	-25.76	3482	0.000	0.133	0.124-0.142	-170.83	3509	0.000
Clu	**Plan**	3.496	3.147-3.925	32.92	14394	0.000	1.419	1.357-1.484	15.89	14395	0.000
	**Clim**	0.605	0.536-0.678	-9.01	9598	0.000	0.612	0.573-0.653	-15.80	9597	0.000
	**Disp**	0.129	0.070-0.189	-20.70	7196	0.000	0.593	0.550-0.637	-15.23	7198	0.000
	**Confl**	0.303	0.216-0.395	-11.31	4797	0.000	0.281	0.237-0.327	-23.42	4796	0.000
Uar	**Plan**	8.825	8.675-8.980	412.99	10707	0.000	2.055	2.055-2.056	6573.65	10667	0.000
	**Clim**	0.449	0.442-0.456	-147.55	7011	0.000	0.586	0.585-0.586	-4342.65	7131	0.000
	**Disp**	0.172	0.168-0.177	-298.53	5330	0.000	0.141	0.140-0.141	-11658.03	5379	0.000
	**Confl**	0.111	0.107-0.115	-387.66	3474	0.000	0.127	0.127-0.128	-12479.05	3537	0.000
Fsy	**Plan**	9.493	8.589-10.608	113.83	14390	0.000	3.788	3.717-3.862	207.46	14396	0.000
	**Clim**	0.322	0.295-0.350	-36.27	9594	0.000	0.398	0.388-0.408	-90.80	9595	0.000
	**Disp**	-0.003	-0.029-0.023	-53.59	7197	0.000	0.045	0.036-0.054	-143.94	7198	0.000
	**Confl**	0.746	0.688-0.807	-7.48	4795	0.000	0.776	0.756-0.798	-18.69	4798	0.000
Cpy	**Plan**	-0.156	-0.21- 0.00	-30.11	10609	0.000	147.984	133.337-166.245	1178.42	10620	0.000
	**Clim**	0.955	0.813-1.105	-0.61	7038	0.545	0.795	0.791-0.800	-87.35	6995	0.000
	**Disp**	0.031	0.000-0.178	-19.20	5259	0.000	0.108	0.104-0.113	-172.71	5319	0.000
	**Confl**	0.372	0.281-0.463	-12.36	3481	0.000	0.156	0.152-0.160	-378.86	3568	0.000

***Gpy**: Galemys pyrenaicus; **Mer**: Mustela erminea; **Mlu**: Mustela lutreola; **Ocu**; Oryctolagus cunniculus; **Asa**: Arvicola sapidus; **Mca**: Microtus cabrerae; **Clu**; Canis lupus; **Uar**. Ursus arctus; **Fsy**: Felix sylvestris; **Cpy**: Capra pyrenaica.*

(it continues in the next page)

		Q3					Q4				
**Sp**	**Factor**	***Peff***	**CI**	**t.test**	**df**	***P***	***Peff***	**CI**	**t.test**	**df**	***P***
Gpy	**Plan**	0.791	0.771-0.811	-18.43	14398	0.000	0.594	0.579-0.608	-47.92	12595	0.000
	**Clim**	6.233	5.937-6.559	145.59	9591	0.000	6.167	5.982-6.364	180.83	8130	0.000
	**Disp**	0.012	-0.004-0.028	-89.08	7092	0.000	0.026	0.010-0.041	-105.96	5855	0.000
	**Confl**	0.146	0.124-0.168	-59.57	4620	0.000	0.083	0.063-0.103	-79.09	3801	0.000
Mer	**Plan**	0.958	0.956-0.960	-41.59	10636	0.000	0.761	0.760-0.762	-401.63	10636	0.000
	**Clim**	0.172	0.171-0.173	-1270.12	6990	0.000	0.156	0.155-0.157	-2069.23	6990	0.000
	**Disp**	0.007	0.006-0.008	-1798.81	5264	0.000	0.002	0.002-0.003	-2878.15	5264	0.000
	**Confl**	0.127	0.126-0.128	-1619.21	3564	0.000	0.078	0.077-0.078	-2789.37	3564	0.000
Mlu	**Plan**	1.689	1.689-1.690	13107.02	10688	0.000	1.085	1.085-1.085	6517.69	10688	0.000
	**Clim**	0.126	0.126-0.126	-31460.93	7034	0.000	0.137	0.137-0.137	-96539.14	7034	0.000
	**Disp**	0.007	0.007-0.007	-41320.29	5317	0.000	0.015	0.015-0.015	-99992.94	5317	0.000
	**Confl**	0.262	0.262-0.262	-28094.61	3504	0.000	0.170	0.170-0.170	-88922.80	3504	0.000
Ocu	**Plan**	0.154	0.152-0.155	-724.59	14398	0.000	0.146	0.145-0.148	-919.63	14398	0.000
	**Clim**	0.573	0.571-0.576	-257.53	9598	0.000	0.516	0.514-0.518	-374.12	9598	0.000
	**Disp**	0.003	0.001-0.005	-668.24	7197	0.000	0.027	0.026-0.029	-803.92	7197	0.000
	**Confl**	0.565	0.561-0.569	-177.74	4793	0.000	0.507	0.504-0.510	-258.42	4793	0.000
Asa	**Plan**	0.319	0.315-0.323	-250.48	14397	0.000	0.249	0.245-0.252	-330.07	14397	0.000
	**Clim**	0.405	0.400-0.410	-172.18	9598	0.000	0.482	0.477-0.487	-169.36	9598	0.000
	**Disp**	0.004	0.000-0.009	-299.37	7198	0.000	0.011	0.007-0.015	-344.18	7198	0.000
	**Confl**	0.382	0.374-0.389	-122.44	4798	0.000	0.311	0.305-0.317	-163.45	4798	0.000
Mca	**Plan**	4.493	4.455-4.531	399.45	10663	0.000	2.934	2.915-2.953	319.53	10663	0.000
	**Clim**	2.213	2.197-2.229	202.42	7099	0.000	2.331	2.316-2.346	238.49	7099	0.000
	**Disp**	1.059	1.049-1.070	11.60	5395	0.000	0.968	0.959-0.976	-7.17	5395	0.000
	**Confl**	0.120	0.111-0.129	-171.95	3509	0.000	0.169	0.160-0.177	-173.90	3509	0.000
Clu	**Plan**	0.680	0.653-0.708	-19.33	14395	0.000	0.434	0.414-0.455	-42.66	14392	0.000
	**Clim**	0.607	0.576-0.640	-20.06	9597	0.000	0.628	0.600-0.657	-21.63	9594	0.000
	**Disp**	0.251	0.210-0.293	-6.59	7198	0.000	0.090	0.048-0.134	4.32	7191	0.000
	**Confl**	0.312	0.276-0.349	-27.42	4796	0.000	0.239	0.208-0.270	-36.26	4772	0.000
Uar	**Plan**	1.295	1.294-1.295	4787.09	10667	0.000	1.030	1.030-1.030	990.51	10667	0.000
	**Clim**	0.465	0.465-0.466	-11285.41	7131	0.000	0.466	0.466-0.466	-18694.36	7131	0.000
	**Disp**	0.182	0.182-0.182	-19177.79	5379	0.000	0.200	0.200-0.200	-28510.72	5379	0.000
	**Confl**	0.076	0.076-0.076	-21734.91	3537	0.000	0.056	0.056-0.056	-31462.96	3537	0.000
Fsy	**Plan**	1.738	1.723-1.754	129.98	14396	0.000	0.852	0.846-0.858	-42.98	14396	0.000
	**Clim**	0.510	0.503-0.517	-108.30	9595	0.000	0.593	0.587-0.599	-109.91	9595	0.000
	**Disp**	0.197	0.190-0.203	-172.71	7198	0.000	0.337	0.332-0.343	-172.54	7198	0.000
	**Confl**	0.568	0.556-0.579	-60.17	4798	0.000	0.489	0.480-0.497	-93.36	4798	0.000
Cpy	**Plan**	27.760	27.271-28.267	1317.39	10620	0.000	7.456	7.409-7.504	927.64	10620	0.000
	**Clim**	0.784	0.780-0.788	-96.08	6995	0.000	0.866	0.861-0.870	-54.91	6995	0.000
	**Disp**	0.209	0.204-0.214	3.48	5319	0.001	0.224	0.219-0.230	8.85	5319	0.000
	**Confl**	0.126	0.122-0.131	-342.45	3568	0.000	0.188	0.183-0.193	-288.93	3568	0.000

Gpy*: Galemys pyrenaicus;* ***Mer****: Mustela erminea;* ***Mlu****: Mustela lutreola;* ***Ocu****; Oryctolagus cunniculus;* ***Asa****: Arvicola sapidus;* ***Mca****: Microtus cabrerae;* ***Clu****; Canis lupus;* ***Uar****. Ursus arctus;* ***Fsy****: Felix sylvestris;* ***Cpy****: Capra pyrenaica.*

Table S5 – Summary of the multivariate distance matrix regression analysis ran over the dissimilarity matrix for the mean number of species (obtained across several conflict levels) within each CCC-cell and time-period. Solutions were obtained varying planning design (Plan), climate scenarios (Clim), dispersal ability assumptions (Disp) and conflict types (Confl).

Factor	Statistic	df	Pseudo R2	*P*
**Plan**	2.01	1	0.430	< 0.002
**Clim**	0.87	2	0.187	< 0.002
**Disp**	0.19	3	0.041	< 0.002
**Confl**	0.60	5	0.128	< 0.002
