# Supplementary material for: A quantitative analysis on the effects of critical factors limiting the effectiveness of species conservation in future time
Source: Ecol Evol. 2018 Feb 24;8(6):3457–67. doi: 10.1002/ece3.3788 (PMC5869367; doi:10.1002/ece3.3788)
Supplement: Supplementary file 4 [file ECE3-8-3457-s004.pdf]

SUPPLEMENTARY MATERIAL

APPENDIX 4

- SOFTWARE INSTRUCTIONS -

A very beta version of the *minShortfall* (and *maxPers*) model

Currently, we are working on the development of a free-accessible, stand-alone software capable to solve the here introduced models using sizable datasets and therefore to provide support for pro-active real-world conservation plans. Meanwhile we retrieve a preliminary software application to run ***minShortfall***, using a linear-decay probability of successful dispersal in function of the Euclidean distance between source and target grid-cells (distancing less than the maximum dispersal distance of each species). Also, in this version time-periods are restricted to four. If less time-periods are to be used (i.e. two or three), data for the remaining periods (third and fourth, or fourth, respectively) should be replicas of the last one. This refers to the cost and climatic suitability data. Budget for the last period should then be distributed among the budgets referenced to the “fake”-periods.

The model is archived at Figshare at: https://doi.org/10.6084/m9.figshare.5700715.v1 under the *SOFTWARE_beta* folder.

The ***maxPers*** to be run for each species may be replicated using ***minShortfall*** with very large species persistence targets (to emulate infinite values) and a large value for budgets (idem).

The tool to formulate and solve the persistence-targets-setting framework is deliverable through the executable file (*2.MIP1*). Before running this executable users are requested to first run other executable (*1.CorridorPoolBuilder*) which identifies, for each species, the set of corridors (CCCs) with the highest persistence scores (corridor pool). Only after having identified the corridor pool, are users able to run *2.MIP1*.

Given that good performance solvers for MIP are not available for free for the general public (although see the free Academic Initiative for Cplex), the MIP executable only formulates the problem into mps files that are readable by several MIP solvers. It is recommended that users choose among the available ones:

- CPLEX
- MATLAB
- R CRAN
- NEOS online platform

For ordinary-sized datasets, the *Rglpk* package in R is also an option.

PREPARING DATA

In *data* folder we put illustrative data referred to 50 virtual species in a map of 1,000 grid cells. If users intend to run their own datasets we suggest them to copy and paste the required data from the respecting files (*T0, T1, T2, T3, DISTMAX and K_S*). Additionally, users should not forget to use the GEOGR file corresponding to the map size.

We invite users not to rename the folders *data* and *output_MIP* as the executables target them by name.

1) To generate the corridor pool (1.CorridorPoolBuilder.exe)

Input

GEOGR.txt	a table with the longitudinal (1st column) and latitudinal (2nd column) coordinates (in degrees) of each grid cell in the map (row wise).
T0.txt	a table with the local (environmental) suitabilities at the baseline period (to) for each species (column wise) for a set of grid cells (row
T1.txt	a table with the local (environmental) suitabilities at T1 period (to) for each species (column wise) for a set of grid cells (row wise)
T2.txt	a table with the local (environmental) suitabilities at T2 period (to) for each species (column wise) for a set of grid cells (row wise)
T3.txt	a table with the local (environmental) suitabilities at T3 period (to) for each species (column wise) for a set of grid cells (row wise)
DISTMAX.txt	a vector with the maximum distance (km/time-interval in yrs) for each species.
K_S.txt	a vector with the size of the corridor pool for each species.
MAXAREA.txt	a value with the maximum number of cells to be part of the final solution.

Output

TABLE.txt	a file with the definition of the corridor pools for each species. Each set of four vertical values represents the areas that define a corridor in each time period (T0-T3). Sets of corridors are defined in sequence (top to bottom) for the species under analysis.
K_S_mod.txt	a vector with the size of the corridor pool for each species (corrected for the case that *K_S.txt* values are unfeasible with data). It corresponds to the number of columns in *TABLE.txt* for each species, respectively.
TIME1.txt	Computational time (in sec).

2) MIP1 implementation (2.MIP1.exe)

Input:

GEOGR.txt	a table with the longitudinal (1st column) and latitudinal (2nd column) coordinates (in degrees) of each grid cell in the map (row wise).
COST.txt	A table with the cost of each grid cell (row wise) in each time period (column wise)
T0.txt	a table with the local (environmental) suitabilities at the baseline period (to) for each species (column wise) for a set of grid cells (row
T1.txt	a table with the local (environmental) suitabilities at T1 period (to) for each species (column wise) for a set of grid cells (row wise)
T2.txt	a table with the local (environmental) suitabilities at T2 period (to) for each species (column wise) for a set of grid cells (row wise)
T3.txt	a table with the local (environmental) suitabilities at T3 period (to) for each species (column wise) for a set of grid cells (row wise)
TABLE.txt	a file with the definition of the corridor pools for each species. Each set of four vertical values represents the areas that define a corridor in each time period (T0-T3). Sets of corridors are defined in sequence (top to bottom) for the species under analysis.
K_S_mod.txt	a vector with the size of the corridor pool for each species (corrected for the case that *K_S.txt* values are unfeasible with data). It corresponds to the number of columns in *TABLE.txt* for each species, respectively.
DISTMAX.txt	a vector with the maximum distance (km/time-interval in yrs) for each species.
MAXAREA.txt	a value with the maximum number of cells to be part of the final solution.
TARGET.txt	a vector with the persistence targets defined for each species.

Output:

MIP1 mps	the formulation to be given to a mps-file-based MIP solver (e.g. Cplex, NEOS platform).
Pers_TAB.txt	A list defining for each species (row-wise) the estimated persistence in the corridors defined in *TABLE.txt*
